# The *Apis mellifera* Filamentous Virus Genome

**DOI:** 10.3390/v7072798

**Published:** 2015-07-09

**Authors:** Laurent Gauthier, Scott Cornman, Ulrike Hartmann, François Cousserans, Jay D. Evans, Joachim R. de Miranda, Peter Neumann

**Affiliations:** 1 Agroscope, Swiss Bee Research Centre, Schwarzenburgstrasse 161, CH-3003 Bern, Switzerland; E-Mails: uli.hartmann@gmx.ch (U.H.); coussera@supagro.inra.fr (F.C.); peter.neumann@vetsuisse.unibe.ch (P.N.); 2 Bee Research Laboratory, Beltsville, MD 20705, USA; E-Mails: scott.cornman@gmail.com (S.C.); jay.evans@ars.usda.gov (J.D.E.); 3 Department of Ecology, Swedish University of Agricultural Sciences, Uppsala 750 07, Sweden; E-Mail: joachim.de.miranda@slu.se; 4 Institute of Bee Health, Vetsuisse Faculty, University of Bern, CH-3001 Bern, Switzerland

**Keywords:** *Apis mellifera*, filamentous virus, genome annotation

## Abstract

A complete reference genome of the *Apis mellifera* Filamentous virus (AmFV) was determined using Illumina Hiseq sequencing. The AmFV genome is a double stranded DNA molecule of approximately 498,500 nucleotides with a GC content of 50.8%. It encompasses 247 non-overlapping open reading frames (ORFs), equally distributed on both strands, which cover 65% of the genome. While most of the ORFs lacked threshold sequence alignments to reference protein databases, twenty-eight were found to display significant homologies with proteins present in other large double stranded DNA viruses. Remarkably, 13 ORFs had strong similarity with typical baculovirus domains such as PIFs (*per os* infectivity factor genes: *pif-1*, *pif-2*, *pif-3* and *p74*) and BRO (Baculovirus Repeated Open Reading Frame). The putative AmFV DNA polymerase is of type B, but is only distantly related to those of the baculoviruses. The ORFs encoding proteins involved in nucleotide metabolism had the highest percent identity to viral proteins in GenBank. Other notable features include the presence of several collagen-like, chitin-binding, kinesin and pacifastin domains. Due to the large size of the AmFV genome and the inconsistent affiliation with other large double stranded DNA virus families infecting invertebrates, AmFV may belong to a new virus family.

## 1. Introduction

**Figure 1:**
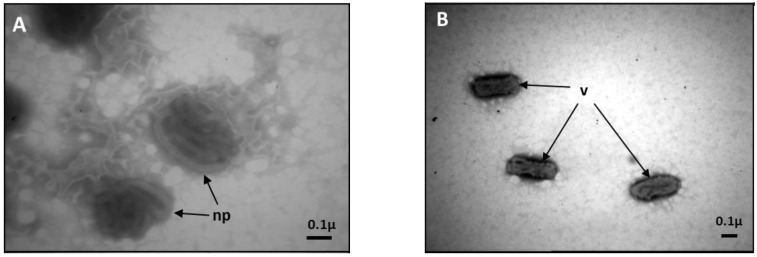
Morphology of AmFV virions: (**A**,**B**) Electron micrographs of “milky” bee hemolymph containing characteristic AmFV nucleoproteins (np) and enveloped virions (v). Approximate size is indicated by scale bars.

*Apis mellifera* filamentous virus (AmFV) was originally described as a rickettsia disease of honeybees (*Apis mellifera*) [1], but was later characterized as a large enveloped DNA virus [2]. It derives its name from the long (3150 × 40 nm) filamentous nucleoprotein that folds in three superimposed figure-8 loops (Figure 1) into a 450 nm × 170 nm rod-shaped virion [2,3]. The nucleoprotein contains a central core of double stranded DNA (dsDNA) wrapped by two major nucleoproteins while the tri-laminate virion envelope contains lipids, two major proteins and several minor proteins [4]. One major diagnostic feature of AmFV infection is that the hemolymph of severely infected adult honeybees becomes milky-white due to cellular degradation and the large number of virions present, which is also a primary feature of insect ascoviruses infecting Lepidopteran hosts [5].

*A. mellifera* filamentous virus epizooties were identified in various parts of the world through electron microscopic examination of bee hemolymph samples [6]. AmFV was shown to multiply in different bee tissues, principally in the fat body and ovarian tissues of adult bees [7]. Highly infected bees display signs of weakness and are usually gathered at the hive entrance. Although it may occasionally induce such overt, colony-level symptoms, AmFV is usually considered a weakly pathogenic virus of honeybees, with low impact on host lifespan [7,8]. Experimental infections can be established by feeding adult bees AmFV particles, but only when co-infected with *Nosema apis* spores [9]. Historically, there has been a strong association between *Nosema sp.* and AmFV infections [7]. AmFV has occasionally been associated with winter honeybee colony mortality [3], but its annual peak incidence is mostly in spring, coinciding with the peak incidence of *N. apis* [9]. Filamentous viruses with similar morphology and tissue distribution have also been observed in a number of parasitic wasps, where they may be associated with super-parasitism behavior [10].

Here we present the complete nucleotide sequence and annotation of the AmFV genome, as well as insights into the distribution of this virus in honeybee colonies. Although some coding sequences are homologous with other large dsDNA viruses of invertebrates, such as baculoviruses, the size of AmFV genome, the proportion of putatively non-coding sequences and the large number of open reading frames (ORFs) lacking database matches all suggest a very distinct virus that merits classification in a new family of insect viruses.

## 2. Materials and Methods

### 2.1. AmFV DNA Isolation, Sequencing, and Assembly

Viral particles were purified from six infected honeybee workers, collected from a single colony in the spring of 2009 in Bern, Switzerland, that were displaying the characteristic “milky hemolymph” AmFV clinical signs. After homogenization in 1.5 mL of 50 mM Tris pH 7.5 buffer, viral particles were separated by centrifugation on a 10%–60% Nicodenz^®^ (Sigma-Aldrich, St Louis, MI, USA) gradient. Two visible bands were extracted from the gradient and analyzed by transmission electron microscopy (TEM). The presence of AmFV enveloped virions (Figure 1) was confirmed in the lower band while the upper band contained mostly degraded nucleoproteins. Total DNA was purified from the lower band by phenol-chloroform extraction and precipitation with ethanol. The DNA was resuspended in 5 mM Tris pH 7.5 and quantified using the Qubit^®^ system (Life Technologies, Carlsbad, CA, USA) One hundred nanograms of DNA were sent to Fasteris Life Science Co. (Geneva, Switzerland) where a paired-end library was prepared using the TruSeq^®^ Nano DNA LT kit (Illumina, San Diego, CA, USA) with bead-based size selection of 550 bp fragments. The library was sequenced on an Illumina HiSeq 2500 Analyzer, producing 100-bp paired reads that were quality filtered and adapter screened by the sequence provider and assembled *de novo* at a range of kmers using the VELVET program [11].

Alignments from the scaffold dataset were produced using the SeqMan (Lasergene 7.0; Dnastar Inc., Madison, WI, USA) software. Gaps and ambiguous regions were amplified by PCR using a high fidelity polymerase (HIFI, Kapa-Biosystems, Wilmington, MA, USA) and appropriate primers. The resulting amplicons were sequenced on both strands using the Sanger sequencing method.

Jellyfish [12] was used to evaluate the heterogeneity of the isolated sample by plotting the coverage of all kmers of length 59 occurring in the raw reads. Batches of kmers were assembled with CLC Genomics v. 7.1 (Qiagen, Valencia, CA, USA) using a kmer of 59 and searched by BLASTX against the UniRef90 database [13].

### 2.2. AmFV Sequence Analysis

Methionine-initiated open reading frames (ORFs) of 100 amino acids or more were identified with the getorf program of the EMBOSS package [14]. Those ORFs with no or minimal overlap were identified using the AMIGENE gene prediction software [15] and were assumed to encode putative proteins. The sequences of these ORFs were compared with BLASTP to the “UniRef90” database and with delta-blast to the conserved domain database (CDD) of NCBI, in both cases with a minimum e-value of 1.0e^−5^, as well as against the Pfam-A database (Hmmer3) with a minimum e-value for the best domain match of 0.1. The graphical representation of genomic ORFs was performed using GenQuest (Lasergene 7.0; Dnastar Inc., Madison, WI, USA). Multiple sequence alignments were performed using the Multalin software [16]. Phylogenetic analyses were conducted using the pipeline offered by Phylogeny.fr: MUSCLE was used for multiple alignment, Gblocks for alignment curation, PhyML for phylogeny and finally TreeDyn for tree drawing [17]. Pfam domains of proteins were identified using SMART [18]. The GC content of the genome was calculated using DNA plotter [19].

### 2.3. Assembly of AmFV-Like Contigs from the USA

Cornman *et al.*, 2010 [20] identified several putative virus sequences from shotgun genomic sequencing of a U.S.A. sample of the bee parasite *Varroa destructor*, which, by BLAST alignment, were found to partly overlap with the AmFV genome sequence presented here. The original reads (N = 95,386) underlying those varroa-derived contigs were reassembled with Mira4 [21], in order to identify and maximize the major contigs for this virus isolate, resulting in 844 contigs of 300 bp or more, with an N50 of 2052 bp. MegaBLAST was used to select a subset of contigs matching the AmFV reference assembly presented here. These 88 contigs totaled 461.7 kb of sequence with an average G + C content of 50.0%. The coverage and consistency of the final genome model was assessed by mapping read pairs with “Bowtie2” using the “very-sensitive” and “end-to-end” quick switches. The statistics for this alignment were calculated with SAMtools [22] and the Picard package [23].

### 2.4. AmFV PCR Detection

**Figure 2:**
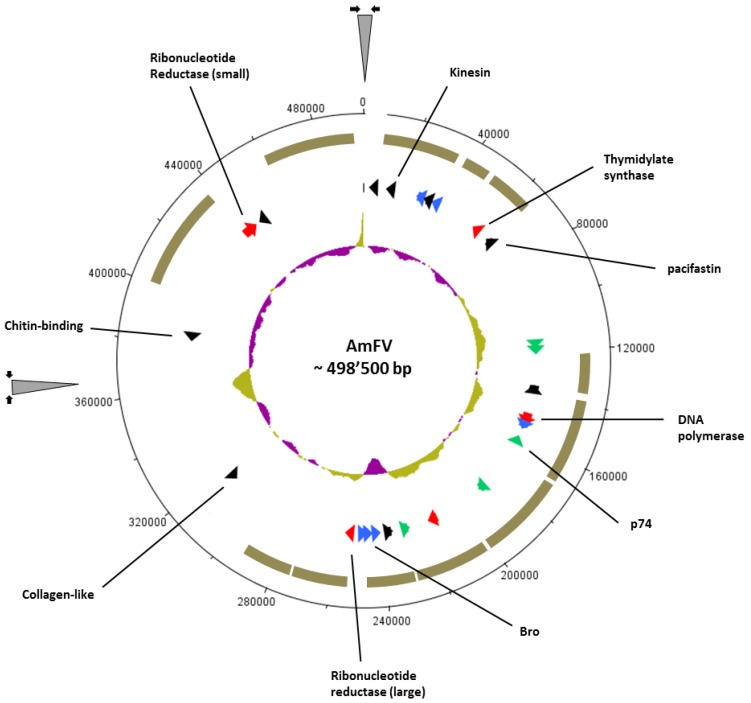
Schematic representation of the AmFV genome. Characteristic ORFs presenting similarities to other large dsDNA viruses are indicated. Colors refer to putative functions: DNA replication and nucleotides metabolism genes (red), *pif* and *p74* homologs (green), *Bro* genes (blue), and putative virulence genes (black). The brown circle fragments correspond to the positions of the larger contigs (>8500 bp) filtered out from *V. destructor* metagenome assembly and assigned initially as baculovirus-like sequences [20]. The inner circle represents the GC plot (above average: gold; below average: purple). The grey triangles flanked by arrows indicate unresolved sequences.

Adult and larval honeybees were collected from their colony and analyzed either individually or in pools of 100 workers. Organs were obtained after dissection in PBS under a stereomicroscope, as described previously [24]. Bee products (honey and pollen) were directly collected from the frames. Bees were homogenized in Tris 20 mM, pH 7.5 – NaCl 400 mM buffer using a 5 mm metal bead and the Tissue Lyzer^®^ homogenizer (Qiagen, Valencia, CA, USA) for 30 s, while bee products and organs were directly homogenized following the same process in a denaturing buffer (Buffer T1, Nucleospin Tissue^®^ kit, Macherey-Nagel, Düren, Germany). Fifty microliters of the homogenate was used for DNA extraction, using the NucleoSpin^®^ Tissue kit (Macherey Nagel, Düren, Germany) following the manufacturer’s recommendations. The DNA was eluted in 100 µL of water and quantified by spectrophotometry (Nanodrop^®^, Wilmington, DE, USA) prior to being used for PCR analysis. The quality of the DNA samples was confirmed by quantifying the *A. mellifera* actin gene in the samples using quantitative PCR as described previously [25]. Diagnostic PCR primers were designed for 3 genes displaying 100% nucleotide identity between the Swiss and US AmFV sequences and located in different regions of the AmFV genome (see Figure 2 and Suppl. File 1): a thymidylate synthase gene (AmFV_28: forward CGCATGTACCAACAACTCGTAC and reverse CACAGTTGGTGTAGCGCAGT, described by Cornman *et al.*, 2010 [20]), a ribonucleotide reductase gene (AmFV_221: forward AGATCGCCCGCTTTGTCGCC and reverse AGCGGGCCTCGGTGTACACT) and a Bro gene (AmFV_112: forward CAGAGAATTCGGTTTTTGTGAGTG and reverse CATGGTGGCCAAGTCTTGCT). PCR reactions were performed using standard reagents containing 0.4 μM of each primer and a cycling profile of 2 min at 94 °C followed by 35 cycles of: 30 s at 94 °C, 30 s at 56 °C and 45 s at 72 °C. The identity of the PCR amplicons was confirmed using Sanger sequencing.

### 2.5. Nucleotide Sequence Accession Number

The nucleotide sequence of the AmFV genome was deposited in GenBank under accession KR819915.

## 3. Results and Discussion

### 3.1. Assembly and Nucleotide Sequence Analysis of AmFV

The sequencing output was 25.67 million read pairs with a median size of 404 bp. Trimming and assembly resulted in the selection of an optimal 93-bp kmer assembly, based on the highest N50 (155,624 bp) and proportion of reads mapping to contigs (90.1%). The resulting 276 contigs had a total length of 3.9 Mbp. Scaffolding by Velvet produced two major scaffolds of 367,058 and 131,360 nucleotides. These scaffolds could furthermore be joined by PCR, such that the junctions between the scaffolds could be sequenced from both directions by Sanger sequencing of the PCR fragments. However, the internal regions of the gaps between the scaffolds, of approximately 250 and 1200 nucleotides, could not be resolved due to their high GC content (>70%) and the presence of repeated sequences. These results led us to infer that the AmFV genome consists of a circular dsDNA molecule of approximately 498,500 nucleotides, with an average GC content of 50.8% (Figure 2). The AmFV genome therefore appears intermediate in size between mimiviruses (>1 Mb) [26] and the White Spot Syndrome Virus (WSSV) [27] or the largest entomopoxviruses [28,29].

A plot of kmer abundance is shown in Supplemental File 2a. Two distinct modes of coverage are evident around 160× and 380×. Based on the inflection points of the curve in Supplemental Figure 2a, kmers in the coverage range of 50–220 and 250–750 were investigated separately as likely deriving from two distinct genomes present in the isolate. The lower coverage mode included approximately four times as many distinct kmers (5,905,345 *vs.* 1,547,736), with a GC content of 36.7% *vs.* 61.7%. Each batch of kmers was assembled separately and aligned with BLASTX to the UniRef90 database (matches summarized in Supplemental File 2b). The results indicate that the first kmer coverage mode represents genomic sequence of an *Arsenophonus* bacterium, because a large majority of best matches were to *Arsenophonus nasoniae*, generally with protein similarities >90%. The second, high-GC kmer population appears to represent a plasmid and/or phage associated with *Arsenophonus*, because *Arsenophonus* is again the most frequently matched taxon, but with a greater representation of other taxa and the spectrum of proteins identified is narrower and dominated by those associated with conjugation, replication, and phage assembly. Similar BLASTX matches as those found for the AmFV genome model were also found in this second pool of kmers, although a minority of matches overall. Thus, the isolation and assembly of AmFV appears to have been complicated by co-occurrence of these other genomes, explaining the need for substantial laboratory confirmation and scaffolding of the AmFV genome model despite high total coverage of paired-end reads. We are nevertheless confident about the accuracy of the assembled AmFV sequence due to the considerable overlap with US contigs isolated from the *V. destructor* metagenome (Figure 2).

### 3.2. Gene Content Analysis

A total of 247 putative non overlapping coding DNA sequences with a minimum length of 100 amino acids were assumed to encode putative proteins. If these 247 represent the total coding DNA of the genome, then the coding density of the *Am*FV genome is about 65%. The coding density value of AmFV is then intermediate regarding values encountered in other large dsDNA viruses, which range for instance between 90.5% (Mimivirus) and 26.9% (Bracovirus) [30]. The predicted ORFs are evenly distributed on both strands and only 40 ORFs (16%) had threshold similarity to proteins in the public sequences databases (see supplementary file S3). Of these, 28 ORFs showed significant homology with proteins of large DNA viruses, 13 of which with baculoviruses [31] (Table 1 and Table S3).

**Figure 3:**
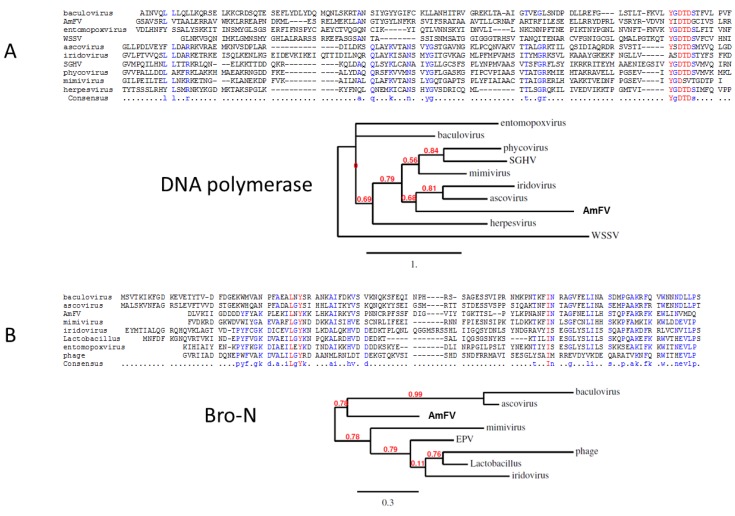
Multiple sequence alignments and phylogenetic trees for the DNA polymerase (**A**) and Bro-N (**B**) domains. Branch support values are indicated in red. GenBank entries from top to bottom: A, gi|365199561|, gi|1063688|, gi|409978642|, gi|589287870|, gi|9964364|, gi|15021419|, gi|187903038|, gi|312233904|, gi|2702251|, and gi|311977705|; and B, gi|325152622|, gi|21668326|, gi|55416627|, gi|9631452|, gi|672972322|, gi|736994060|, and gi|660515707|.

The most conserved ORFs in terms of percent amino-acid identity are those associated with nucleotide metabolism, e.g., AmFV_116 and AmFV_221, encoding putative subunits of a ribonucleotide reductase. This enzyme is highly conserved among living organisms and catalyzes the formation of deoxyribonucleotides from ribonucleotides for the synthesis of DNA. Other highly conserved genes are those involved in DNA synthesis, such as AmFV_28 encoding a putative thymidylate synthase, and AmFV_76, encoding a putative 1954 amino acid DNA polymerase characterized by a type B DNA polymerase (POLBc) domain typical of large dsDNA virus families (Figure 3A).

**Table 1:** Putative homologies of methionine initiated ORFs, with a minimum length of 100 amino acids, identified throughout the AmFV genome (best match against Pfam-A database with Hmmer3 (**left**) and corresponding best BLASTP match against viral sequences (**right**)). Records with e-value under threshold are indicated with a dashed line. The percent identity at the amino-acid level between Swiss and US protein sequences are noted on the right.

	Best Pfam-A database match with HMMer3 (0.1 cut off)					Best match with viral sequences database (BLASTP search taxid 10239, 1.0e-5 cut off)					AmFV (USA) % identity
Putative function	*Am*FV ORF	Size (aa)	Pfam code domain	e-value	Pfam n°	CDS	e-value	Length / %similarity	Score	Accession number	
*DNA replication and nucleotide metabolism*	AmFV_76	1954	DNA_pol_B	6.5e-17	PF00136.16	-	-	-	-	-	98.1
	AmFV_97	1602	DNA ligase	2.8e-08	PF04675	Hyp. protein [*P. bursaria* Chlorella virus AR158]	4.0e-12	509 / 53% (61/115)	72	YP_001498814.1	93.7
	AmFV_116	878	Ribonuc_red_IgC	5.9e-180	PF02867.10	RR1 [*S. litura* nucleopolyhedrovirus]	0.0	770 / 42% (521/883)	711	NP_258291.1	99.7
	AmFV_221	2332	Ribonuc_red_sm	3.4e-96	PF00268.16	RR2B [*S. litura* nucleopolyhedrovirus]	7.e-56	333 / 72% (132/182)	203	NP_258331.1	97.8
	AmFV_28	610	Thymidylat_synt	2.0e-86	PF00303.14	wsv067 [Shrimp white spot syndrome virus]	3.0e-82	289 / 63% (188/294)	265	NP_477589.1	98.4
*Virion structure and morphogenesis*	AmFV_125	506	Capsid_NCLDV	1.5e-02	PF04451.7	Hyp. protein [Organic Lake phycodnavirus]	5.0e-10	238 / 40% (116/283)	65	ADX05786.1	99.4
	AmFV_102	401	Baculo_44	1.3e-16	PF04631.7	PIF-2 [*G. bimaculatus* nudivirus]	2.0-15	378 / 48% (84/173)	82	YP_001111333.1	99.8
	AmFV_90	279	Pif-3	4.0e-06	PF05006.7	PIF3 [*E. ello* granulovirus]	9.0e-06	188 / 39% (64/164)	50	YP_009091870.1	99.6
	AmFV_61	1057	Pif-1	1.6e-17	PF05092.7	PIF1 [*S. littoralis* nucleopolyhedrovirus]	3.0-13	525 / 43% (99/230)	77	AGE89974.1	95.7
	AmFV_62	829	Pif-1	5.8e-25	PF05092.7	DekiORF31 [*D. kikuchii* nucleopolyhedrovirus]	9.0e-19	536 / 48% (109/226)	95	AFS51909.1	98.6
	AmFV_79	1196	Baculo_p74	6.6e-10	PF08404.5	P74 [*B. mori *nucleopolyhedrovirus]	1.0e-9	645 / 50% (72/144)	68	NP_047536.1	96.8
*Unknown*	AmFV_110	626	Bro N	1.82e-05	PF02498	BRO-C [*M. configurata* nucleopolyhedrovirus]	8.0e-11	326 / 50% (110/219)	69	NP_689249.1	96.9
	AmFV_112	498	Bro N	1.5e-13	PF02498.12	BRO-B [*C. chalcites* nucleopolyhedrovirus]	7.0e-17	628 / 48% (134/279)	85	YP_249673.1	97.8
	AmFV_108	662	Bro N	1.7e-09	PF02498.12	BRO-M [*L. xylina* nucleopolyhedrovirus]	2.0e-12	474 / 46% (95/204)	75	YP_003517887.1	99.2
	AmFV_17	1313	Bro N	6.5e-09	PF02498.12	DekiORF51 [*D. kikuchii* nucleopolyhedrovirus]	8.0e-08	480 / 49% (55/112)	62	AFS51929.1	90.9
	AmFV_9	181	ns	ns	ns	BRO-D [*C. chalcites* nucleopolyhedrovirus]	7.0e-07	429 / 56% (42/75)	53	YP_249718.1	100
	AmFV_77	432	ns	ns	ns	BRO-6 [*S. litura* granulovirus]	1.0e-05	485 / 42% (58/136)	52	YP_001257066.1	98.5
	AmFV_197	898	Chitin binding 3	6.8e-25	PF03067.10	-	-	-	-	-	96.1
	AmFV_104	596	Collagen	2.2e-07	PF01391.13	collagen repeat [Bacillus phage phBC6A52]	9.0e-07	536 / 60% (46/76)	57	NP_852574.1	94.0
	AmFV_69	422	ns	ns	ns	collagen-like protein [*A. polyphaga* mimivirus]	3.0e-07	1392 / 54% (54/99)	58	YP_003987190.1	95.9
	AmFV_36	501	Pacifastin I	1.6e-10	PF05375.8	-	-	-	-	-	94.3
	AmFV_3	963	Abhydrolase_3	1.3e-09	PF07859.8	-	-	-	-	-	96.7
	AmFV_6	1354	Peptidase_M10	1.2e-10	PF00413.19	-	-	-	-	-	92.2
	AmFV_223	627	Peptidase_M10	1.3e-10	PF00413.19	-	-	-	-	-	97.9
	AmFV_12	576	Kinesin	1.1e-67	PF00225.18	-	-	-	-	-	99.8

Also identified in the AmFV genome were several putative orthologs of the baculovirus *pif-1*, *pif-2*, *pif-3* and *p74* genes. These genes belong to baculovirus core gene set and are required for oral infectivity [31]. They are also present in other DNA viruses infecting arthropods, such as nudiviruses [32,33], salivary glands hypertrophy viruses (SGHVs) [34,35], WSSV [27] and bracoviruses [36,37,38]. It is not known whether these gene families are required for infectivity in these other viral groups, nor whether they were acquired horizontally or instead reflect common ancestry [35]. *Pif* genes are absent from entomopoxvirus genomes, however, even though these viruses are also orally transmitted [39]. Other elements typical of baculoviruses that were also found in the AmFV genome are the *Bro* genes, of which at least six were found in the AmFV genome. BRO proteins, whose functions are still unknown, contain a DNA binding domain and belong to a multigene family that is wide spread among large insect viruses, including entomopoxviruses and iridoviruses, as well as in dsDNA phage and prokaryotic class II transposons [40,41].

Several additional potential virulence factors were identified in the AmFV genome. Such factors encompass a range of functions that affect infectivity and host-virus interactions and often include degradative enzymes (chitinases, proteases) that are common in many insect virus genomes [42]. The AmFV_197 encodes a chitin-binding domain (although it lacks a domain for the corresponding chitinase enzyme) that may play a role during the early infectious process. Chitin-binding domains are also found in the fusolin proteins of entomopoxviruses and baculoviruses that facilitate the passage of the virus through the peritrophic membrane in the gut [43,44] and similar genes are also present in bacterial pathogens of honeybees [45]. The AmFV_36 protein contains four pacifastin motifs, as well as a transmembrane domain. Pacifastin belongs to a family of serine proteinase inhibitors that is found in arthropods but so far not in viruses. It has recently been shown that pacifastin regulates the prophenoloxidase cascade in crabs [46]. This class of serine inhibitors also seems to be involved in regulating certain physiological processes in insects [47]. If the AmFV pacifastin has a similar function, it may interfere with the immune system of its host *A. mellifera*. AmFV_6 and AmFV_223 are proteins with zinc-dependent metalloprotease domains. AmFV_6 is a putative ortholog of a metalloprotease found in *Pieris rapae* granulovirus. Metalloproteases feature often as virulence factors for many pathogens, such as the bacterium *Paenibacillus larvae*, the causative agent of American foulbrood disease in honeybees [48]. AmFV_12 has homology with a domain present in the drosophila kinesin gene (e-value: 2.e^−42^, see Supplementary File S3). Kinesin belongs to a class of motor proteins that has been shown to facilitate intracellular trafficking of viral components along the microtubule cytoskeleton [49,50]. The presence of a putative homologue of an insect cellular gene in the AmFV genome suggests that AmFV may have evolved a mutualistic association with its host, involving horizontal transfer of genes between host and virus, as it is known to occur with certain other large DNA viruses, such as Bracoviruses [37,51], Baculoviruses and Poxviruses [41]. This possibility requires more detailed investigations. There are also several ORFs that contain collagen-like domains. Such domains are found in a variety of large nucleocytoplasmic DNA viruses and may be associated with virulence. It has been shown that collagen repeated sequences are a key determinant of herpes transforming activity [52] and that collagen is expressed at the surface of the mimivirus, a large DNA virus infecting amoebae, and that this can induce an immune response in mice [53].

### 3.3. Sequence Variation between European and North American AmFV Isolates

The baculovirus-like contigs identified in *V. destructor* by Cornman *et al.*, 2010 [20] showed very high nucleotide identity with the AmFV genome sequence from Switzerland, as illustrated in Figure S1. A majority of the ORFs were also identified in this set of varroa-derived contigs from USA, most of them with very high identity scores (Table 1). For example, the putative DNA polymerase was highly conserved between the two AmFV isolates, with just 17 substitutions and six insertions/deletions along the 1954 amino acid sequence, in addition to differences in the number of repetitions of GN/S residues that may represent a hinge region between different domains of the polymerase. These data strongly suggest that the sequences from the USA and Switzerland belong to the same virus, AmFV. One significant difference concerns the putative reverse transcriptase identified by Cornman *et al.*, 2010, indicative of the presence of a retrotransposon, which was absent in the AmFV genome from Switzerland. Mobile elements are rare in large DNA viruses and have so far only been reported in bracoviruses belonging to the *Cotesia* and *Glyptapanteles* genera [54,55] and the *Melanoplus sanguinipes* entomopoxvirus [56].

### 3.4. AmFV Classification

**Figure 4:**
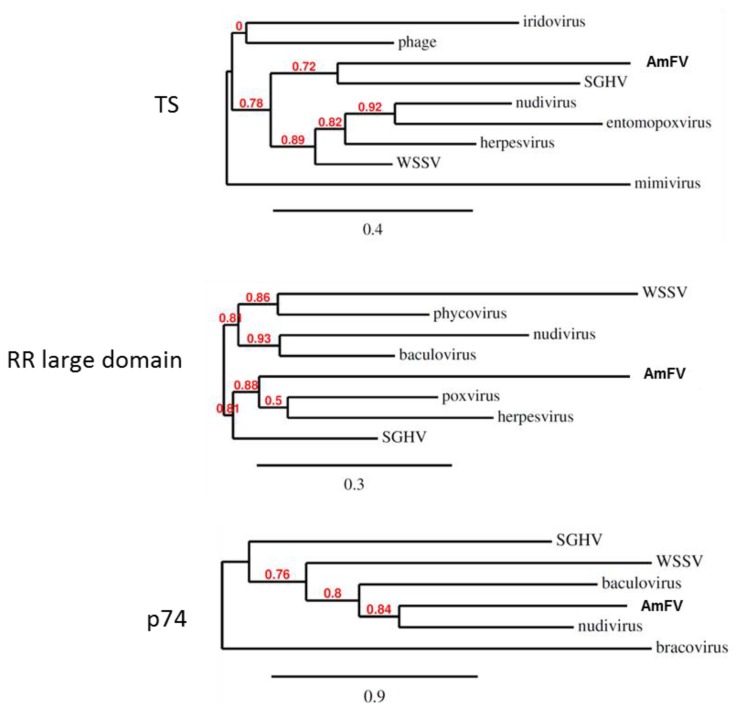
Phylogenetic analyses. Threes were built from thymidylate synthase (TS), ribonucleotide reductase (RR) and p74 proteins. Branch support values are indicated in red. GenBank entries from top to bottom: TS, gi|589287845|, gi|485725392|, gi|187903049|, gi|370702981|, gi|9631429|, gi|551484996|, gi|417072295|, and gi|55417112|; RR large domain, gi|15021484|, gi|347481982|, gi|370702993|, gi|294471329|, gi|22164662|, gi|422933669|, gi|187903102|; p74, gi|187903076|, gi|17016513|, gi|9630072|, gi|370703052|, and gi|531034105|.

The analysis of the AmFV genome sequence highlighted a number of features that make it difficult to assign it to a particular group of viruses. The presence of putative orthologs of baculoviruses suggests a relationship between AmFV and *Baculoviridae* (Table 1). However, this overlap is restricted to a few genes and the large size of the genome and the presence of a high proportion of non-coding sequences are inconsistent with a baculovirus designation. In addition, many other coding sequences present in the AmFV genome, such as the genes involved in DNA replication, display greater similarities with their homologues in other large DNA viruses such as *Poxviridae*, *Herpesviridae* or *Phycoviridae* (Figure 3 and Figure 4). For instance, the phylogenetic analyses of the BroN domain of the AmFV_112 ORF (Figure 3B) and the p74 protein (Figure 4) positioned AmFV close to the baculoviruses, while the DNA polymerase domain of AmFV_76 placed it within the ascovirus and iridovirus cluster (Figure 3A). Likewise, the ribonucleotide reductase IgC domain of AmFV_116 ORF clustered with poxviruses and herpesviruses (Figure 4). This pattern is consistent with networks of multiple horizontal gene transfers (HGT) between viruses infecting the same host, or gene acquisition from the host or its microbiota [41,57]. Indeed, many genes in the AmFV genome have putative viral or bacterial homologues, such as the Bro genes, while others are homologues of insect genes, such as the AmFV_12 ORF which has a kinesin domain also present in *Drosophila* *pseudoobscura*, and the AmFV_36 ORF which has homologies with the precursor of pacifastin venom protein 1 of *Nasonia vitripenis* (Table S3, nr database). The dispersion of core genes throughout the AmFV genome also support potential HGT processes, as does the presence of a retrotransposon in the US AmFV isolate, since such mobile elements are known to be frequently involved in the assimilation of exogenous genes [57,58,59]. The variable GC content observed throughout the AmFV genome (Figure 2) is also consistent with putative HGT processes that may have been responsible for the still unclear taxonomic position of the AmFV genome.

### 3.5. AmFV Distribution in Honeybee Colonies

**Figure 5:**
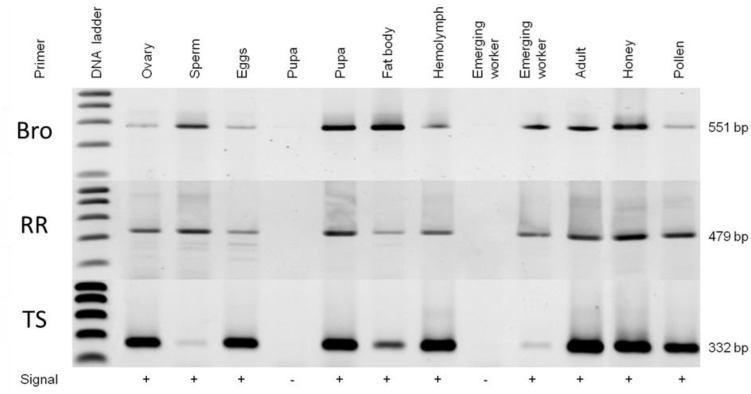
Agarose gel electrophoresis showing AmFV PCR amplicons produced from different individuals (larvae, pupae or adults bees), queen bee tissues, drone sperm or bee products, using three different primer pairs (the thymidylate synthase (TS) primers were described by Cornman *et al.* (2010) [20]).

A PCR-based diagnostic assay was developed from three coding sequences with high nucleotide identity between the AmFV isolates from Switzerland and USA: (a) the putative thymidylate synthase gene (AmFV_28), (b) one of the Bro genes (AmFV_112) and (c) the ribonucleotide reductase gene (AmFV_221). The alignments between the Swiss and US AmFV isolates for these sequences are provided in Figure S1. Using these PCR assays, pools of bees from different colonies were analyzed to compare the prevalence of AmFV in the USA and Switzerland. AmFV was detected in 64% of the Swiss colonies tested (N = 25) and in 100% of the US colonies (N = 15). These results are consistent with classical reports of a relatively high prevalence and wide geographic distribution of AmFV [6,60]. A second screening was conducted to determine the distribution of AmFV in different honeybee organs and bee products found in the colony (Figure 5). The detection of AmFV in pupae, emerging workers and adults suggests that AmFV can infect both the brood and adult stages. Moreover, AmFV infections seem to remain through metamorphosis, thereby suggesting that the virus is not restricted to the alimentary tract. This is supported by the detection of AmFV in the hemolymph of adult workers. AmFV was furthermore also detected in honey and pollen, indicating a possible oral-fecal transmission route. The queen ovaries, eggs and sperm from the drone endophalli were also positive for AmFV, which is indicating considerable potential for either vertical (transovarial) or venereal transmission of this virus, similar to deformed wing virus [61]. This is also supported by the high prevalence of AmFV in queens (11/13 mated queens analyzed were found positive). Likewise, AmFV seems to be present in winter bees, as 12/19 individual overwintering bees from California and Florida were found positive. There was no difference in AmFV prevalence between colonies affected or not affected by Colony Collapse Disorder (CCD), with a combined prevalence of 84% (N = 75). AmFV could only be detected in 3/43 individual *V. destructor* mites and 3/29 pooled mite samples (100 mites/sample), concurring with prior assessments [20]. Moreover, AmFV was also highly prevalent in *V. destructor*-free colonies from the island of Ouessant, France (N = 15), indicating that AmFV presence and prevalence are not closely related to *V. destructor* mite infestation of honeybee colonies. These data suggest that *V. destructor* is probably not a major factor in AmFV prevalence and transmission. The presence of AmFV in *V. destructor* mite samples, even with high abundance, can easily be explained by passive acquisition, since AmFV sheds large amounts of particles and nucleoproteins into the bee hemolymph (“milky hemolymph” symptom), on which the ectoparasite mite feeds. The frequent detection of AmFV in bee guts and food products suggests that oral transmission may be important, consistent with classical observations [7] while the high frequency of AmFV in mated queens, ovaries and sperm suggests a possible vertical transmission route, although previous studies failed to show vertical transmission from artificially AmFV-infected queens [2].

## 4. General Conclusions

We have presented a model of the AmFV genome based on deep sequencing of an enriched extract of semi-pure filamentous virus, identified by classical symptoms, supplemented by Sanger sequencing of specific fragments bridging different assembly scaffolds. Paired-end reads strongly support the accuracy of the final genome model, although they were not able to bridge the long, GC-rich repetitive region that was ultimately scaffolded by PCR. Despite its large size, the presence of a large proportion of novel protein sequence, and its relatively low gene density, AmFV is well conserved at the nucleotide level between two isolates from different continents. This conservation may reflect the high fidelity of the AmFV DNA polymerases and/or the facilitated dispersal of AmFV by the global bee trade. The current genome model can be used as a foundation for functional studies, assays development, quantitative assessments with qPCR, and comparative genomics through metagenomic sequencing of other bee samples.

## Supplementary Information

**Figure S1.** Alignment of three AmFV genes used for designing PCR primers. AmFV-US sequences were identified in the USA by Cornman *et al.*, 2010 [20]. Primer locations are underlined.

**Figure S2.** Details about NGS sequencing data.

**Figure S2a.** The number of unique 59-bp kmers occurring in the filtered reads (vertical axis) is plotted as a function of the number of times that they are present (horizontal axis). As different genomes in a heterogeneous mixture are often present in distinctly different copy numbers, these kmer plots and subsequent analysis of the properties of kmers at different coverage levels allows a partial disentangling of those genomes. Stochasticity, sequence similarity, polymorphism, extraction/sequencing bias, and repetitive components of genomes also influence the abundance of kmers and thus not all kmers are expected to occur proportional to the number of copies of the genome from which they derive.

**Figure S2b.** In total, 59-bp kmers in the coverage range of 50–220 and 250–750 were identified using the program jellyfish and assembled with CLC Genomics v 7.1 at kmer = 59. Best BLASTX matches of the resulting contigs at an e-value threshold of e-10 are shown.

**Table S3.** Blastp hits against (**a**) the non-redundant protein sequences database (http://blast.ncbi.nlm.nih.gov/); (**b**) same as (**a**) but restricted to viruses (taxid 10239); and (**c**) to the UniRef90 database [13]. A list of the AmFV ORFs with corresponding coding sequences is also provided in this file.

**Figure S4.** Multiple amino-acid sequence alignments of thymidylate synthase (TS), ribonucleotide reductase large fragment (RR1), and p74, using the Multalin software [16].
