# Supplementary material for: The Apis mellifera Filamentous Virus Genome
Source: Viruses. 2015 Jul 9;7(7):3798–815. doi: 10.3390/v7072798 (PMC4517127; doi:10.3390/v7072798)
Supplement: Supplementary file 1 [file viruses-07-02798-s001.zip › FigureS4_alignements_proteins.pdf]

Multiple alignements

Thymidylate synthase (AmFV_28)

Go directly to Alignment

```
Multalin version 5.4.1
Copyright I.N.R.A. France 1989, 1991, 1994, 1996
Published research using this software should cite
Multiple sequence alignment with hierarchical clustering
F. CORPET, 1988, Nucl. Acids Res., 16 (22), 10881-10890
Symbol comparison table: blosum62
Gap weight: 12
Gap length weight: 2
Consensus levels: high=90% low=50%
Consensus symbols:
 ! is anyone of IV
 $ is anyone of LM
 % is anyone of FY
 # is anyone of NDQEBZ

 MSF:    380    Check:   0          ..
 Name: AmFV           Len:   380  Check: 4788  Weight:  1.08
 Name: WSSV           Len:   380  Check:  244  Weight:  0.23
 Name: herpesvirus    Len:   380  Check:  316  Weight:  0.23
 Name: nudivirus      Len:   380  Check: 1060  Weight:  0.44
 Name: entomopoxvirus Len:   380  Check:  655  Weight:  0.67
 Name: iridovirus     Len:   380  Check: 2222  Weight:  0.88
 Name: SGHV           Len:   380  Check: 3859  Weight:  0.94
 Name: phage          Len:   380  Check: 1022  Weight:  1.17
 Name: mimivirus      Len:   380  Check: 4179  Weight:  1.40
 Name: phycovirus     Len:   380  Check: 9399  Weight:  2.95
 Name: Consensus      Len:   380  Check:  579  Weight:  0.00

//

            1                                                                                                                                      130
      AmFV                                                                    DRMYQQLVRK VLKRGEQRDD RTGTGTRALF GELLEFDLRG NRLPLLNCRR LSFKSILEEL LWFLRGETDS
      WSSV                                                                MEG EHQYLNLVRE ILERGVKKDD RTGTGTLSIF GPQMRFSLRD DTIPVLTTKK IFWRGVVEEL LWFIRGNTDA
herpesviru  MPEGSTGRPA CLGEQAGSGG DRGRRGRRRR RRGRADARSY LAACRALPLP ADADRPVNPG ERLYLGQLCH ILGHGIERAD RTGVGTLSVF GMHARYSLRG E-FPLLTTKR VYWRGVVEEL LWFIRGCTDA
 nudivirus                                                             MQTNWE EHQYLNTIRS IIETGNRRMD RTKVGTLSHF GVMHRYSLRD NNLPLLTTKT VFVKGVIEEL LWMISGSTNA
entomopoxv                                                            MEITSSD EYQYLKGISE ILSNGRITKN RTGVDTKTVY GLTMKYKL-D DSFPLFTTKS MFWRGIVEEL LWFISGSTNT
iridovirus                                                                  M EENYLNLVNE VIQTGDYRVD RTKIGTFSLF GKQLEFDLSG GTIPLLTTKK IVYTNILKEL LWVIKGSTDS
      SGHV                                                             MLRLTG ERGYLTLVRR VLRDGTLKSN RTGVDTKYIF GDQLRYDLRD NRIAMLTTKK VFFRSALEEL LWFLRGDTNQ
     phage                                                               MTHA DLEYLNLVKK IVKEGHKKED RTGTGTISLF GPQMEFDLSK G-FPLLTTKK LPFRIIAEEL LWFIKGDTDL
 mimivirus                                                                    ENQYIELVKT IMEKGIVKQT RNSI-TKSIF GYQLKYDLSK G-YPIQTIKR SYPKAIFEEL MWMIRGQTDV
phycovirus              MSAKLISVS KPVIEGVNTA EQLIAYAARV SNPENQINNK TAAGLLKYCI RHKHWSIFEQ AFMTLELKTS R-GIAAQVLR HRSFHFQEFS QRYASVIEIP TPQQARFQD- --HTNRQNSL
 Consensus              ......... .......... .......... .......... .......... ...y..lv.. ....g..k.. Rtgi.t..lf g....%dl.. ...p..t.k. .....i.##l lw.i.g.td.

            131                                                                                                                                    260
      AmFV  KILERRGVHI WTEHATRDVL TKRGFPSRRE GDIGPLYGFQ WRFAGLRYTN CEANYAAQKR TRGYDQIAYI LDELTARPHS RRILLNSWNV ADIAEMVLPP CHVMFQLFA- -------DNA -NELSGMLYS
      WSSV  KELAKKKIHI WTANGSREFL DSRGLYDRAE GDLGPVYGFQ WRHFGAEYDT CSSDYTGK-- --GIDQLANI LKTLRENPDD RRMIMTAWNP MDLHLMALPP CHMTAQFYV- -------ANG -E-LSCQLYQ
herpesviru  RRLSERGVKI WNANGSKEFL TARGLGHRRE GDLGPVYGFQ WRHFGATYED ADADYSGR-- --GVDQLAYI LDLIKNHPCD RRMVMSAWNP VDIPKMALPP CHVLCQFYV- -------SAG -E-LSCQLYQ
 nudivirus  KELSRKGVHI WSGNSSRSVL DSLGFTDRQE GDLGPVYGFQ WRHSGTKYVD CETDYSGQ-- --GVDQLQTI IDTLKTNPTD RRIVMCSWNP ADLHQMALPP CHCLAQFYS- -------YDG -K-LSCLMYQ
entomopoxv  NILKNKGIHI WDENGSLKYI KKRGFNDRKE GDLGPVYGFQ WRHFGAKYID MDTNYTNQ-- --GFDQLTHI INKIKNKPES RNIILTAWNP IDVENMVIPP CHCFVQFHV- -------INN -KYLTCQMYQ
iridovirus  KQLNDIGVKV WNDNGSREFL DLCGFTERKE GDLGPIYGFQ WRHAGAQYID CDTDYTGQ-- --GVDQLSQT INTLHNNPEN RRILINSWDV PQLDKMVLPP CHTLVQFYV- -------RKK -SILDCQLYQ
      SGHV  ANLAAKGVNI WRGNSTREYL DSRKLPYPEH ESLGPIYGFQ WRHFGARYID CHTDYTGQ-- --GVDQLAQC MHQIQYEPSS RRMIMTAWNP NDLDKMVLPP CHMSIQFSV- -------NEK LGELSGHMTQ
     phage  KTLLDKNIHI WTPDAYRFYR ESGGTLSYE- -----VFVEK AKEDGFDLGP IYGAQWGRWG DKNIDQLGYV IEEIKNNPDS RRMLVSTWNP TDLSKMALPP CHTMFQFYV- -------SS- -GELSCKLYQ
 mimivirus  SILQKKGVHV WDKNSSKDFL SKYNLP-YEE GDIGPGYGFQ MRYWGAEYTD CKTSYQGQ-- --GIDQLNKC IESIQNNPHD RRIMINLWNC SDLDKMALAP CHFCYMFGVD LYEVPTTSGK KGRLNCHLVQ
phycovirus  DTVSEEEQTW WNTEQEKLYT QSVDLYNKA- --LEKGIAKE CARFVLPLST TTTIYMSG-- --TIRDWIHY IELRTANGTQ KEHIELANAC KDIFVEEFPN IAKALDWI
 Consensus  ..l...g.h. W.......yl .s..l..... ..lgp.ygfq .r.fg..y.. ..t.y.gq.. ..gid#l... ie....np.. rr....awn. .Dl..m.lpp ch...qf.v. .......... ...l.c.l.q
```

```
           261                                                                                                                 380
      AmFV RSADIGLGLP FNIASYAILL HAIAKKVGRT AKTLKVIIGD AHIYNDHIDA --MTEIAARE PRPSPRVYID SRSFDKPLHE YSTDF----- ---FHLFNYH PHEP-VRMRV SV
      WSSV RSGDVGLGVP FNIASYSLLT HLMASMVGLK PGEFILTLGD AHIYNTHIEV --LKKQLCRA PRPFPKLRI- -LMAPEKIED FTIDM----- ---FYLEGYQ PHSGNLQMKM AV
herpesviru RSGDMGLGVP FNIASYALLT YMIAHVSGLR PGELIHVLGD AHIYKNHIEA --VKVQLSRE PRPFPRLHI- -VRTVSSIED FTVDD----- ---FSLEGYD PHPA-IRMDM AV
 nudivirus RSADMGLGVP FNIASYSMLT HMIAHITGLK AHEFVHTIGD AHVYLNHIEP --LKVQLERE PRPFPKLEF- -ARKVENIDS FKYED----- ---FIIKGYD PYPK-IPMVL AV
entomopoxv RSADMGLGVP FNVASYSLLT YMIAH-TNLK PYEFIHNIGN AHIYVNHING --LKKQLERT PNPFPKLKI- -KRQVDNIDD FKFDD----- ---FELIDYN -HLGKIKLDM VV
iridovirus RSADLGLGVP YNIGFYSFLT LVLSKWCNLT PGKFIHTFGD VHVYSNHVEP --LKVQLKRK PYSFPNVKFI GLFSLNDLDN QSLQECCSEW CNSFKVENYT THPF-IKMQM AI
      SGHV RSADLMLGVP FNLVSYSLML HILAHKFGLK AGELVTSYGD LHIYVPHIKN --AMRQVSRL PYASPQIEL- KFGRDKRVEE LTVDD----- ---FAVKNYR HHSQ-LEYEM AA
     phage RSGDAFLGIP FNIASYALLT HIIAKMTGLK VGKFIHTLGD AHLYSNHLDS --AFEQLKRE PRELPKLEV- -KTIHEDIRD YTIDD----- ---FELIGYD PHPV-IKGKL SVGLKDGGEK
 mimivirus RSWDVLLG-- WNTTTAALLT YLIANHCDLD PGILVHSISD AHIYQSHIDS GAISQLLQRK CRKFPNLVI- -RNKKEKIDD YEFDD----- ---LIIENYY PCPS-ISAEM IA
phycovirus
 Consensus rs.d..lg.p .n...y..lt ..ia....l. ........gd ah.y..hi.. ......l.r. p...p..... .......... ...d.          f....y. .h.. ..... ..........
```

Ribonucleotide reductase IgC domain (AmFV_116)

Go directly to Alignment

```
Multalin version 5.4.1
Copyright I.N.R.A. France 1989, 1991, 1994, 1996
Published research using this software should cite
Multiple sequence alignment with hierarchical clustering
F. CORPET, 1988, Nucl. Acids Res., 16 (22), 10881-10890
Symbol comparison table: blosum62
Gap weight: 12
Gap length weight: 2
Consensus levels: high=90% low=50%
Consensus symbols:
 ! is anyone of IV
 $ is anyone of LM
 % is anyone of FY
 # is anyone of NDQEBZ

 MSF:   1027    Check:   0          ..
 Name: AmFV           Len:  1027  Check: 7111  Weight:  2.07
 Name: baculovirus    Len:  1027  Check: 6582  Weight:  0.31
 Name: SGHV           Len:  1027  Check: 5437  Weight:  0.31
 Name: phycovirus     Len:  1027  Check: 6171  Weight:  0.59
 Name: herpesvirus    Len:  1027  Check: 4830  Weight:  0.66
 Name: nudivirus      Len:  1027  Check: 7544  Weight:  0.74
 Name: poxvirus       Len:  1027  Check: 7187  Weight:  1.52
 Name: WSSV           Len:  1027  Check: 8143  Weight:  1.81
 Name: Consensus      Len:  1027  Check: 2184  Weight:  0.00

//

           1                                                                                                                    130
      AmFV
baculoviru     MSYDHV IKRDGRKEAV FMYKIKCRIE KLCY----GL DMNFVNPTSV AVRVDQGIYS GVTTAELDNL IAETAAAMTI DHSDYSLLAA RLAVSNLHKE TKDSFFDVII DMYEAIDPKT GVKTPMISNF
      SGHV MLPNNTKLFV TKRGGFVQDI VFDKIRKRIE SLCY----NL NADYVHPSTI VVKVVAGLYP GVTTAELDTL AAETAASMMT LHPDYGVLAA RIAISNMHKQ TDDSFARTMK ELYAA----- ----GLISMF
phycovirus        MEV IKRNGTSESV QLDKIMKRLK NLSY----GL D--HVDPAVV SIKVVQGLYD GVTTVQLDNL AVETATYLTM THPDYSKLAA RIAVSSLHKM TSNKFSSVME SLYNYVNTKT GQWSPLIADD
herpesviru    MDSAMTV IKRDGSVVPV DIGKVAERVR KC-------- EIDSVDCELL AQQVQAGIHN GCKTAEIDVL LAHTAASMTT KHPNYGLLAA KVSVSNLHLQ TVPEFSKTCE KLWKNINPDL DVEAPLISDE
 nudivirus   MSQNPRMI VKRNGSVERF DPSKLYARIE RCMTMSEPRL TTAFINIAGI VADVEKGLYD RVSTLELDTL LSETCASMST AHPDHSILAA RIAMQALHKR TRSKFSETIR DLYE------ ---ANIVPQY
  poxvirus
      WSSV
 Consensus   ........ .......... .......... ....    .. .......... .......... .......... .......... .......... .......... .......... .......... ..........

           131                                                                                                                  260
      AmFV                                                                                                           LAS CYLLTISEDS IQGIFETLSR CAFVSQSSGG
```

```
 baculoviru  HYNIIAANAY RLNSAIVHDR DF-NYDYFGF KTMQRSYLFK INGVTV-ERP QYMMMRVAIG IHGEDIDAAI DTYNLMSNGY FTHASPTLFS AATPKAQMSS CFLVATKEDS IEGIYDTLKQ CAMISKSGGG
       SGHV  HYDVVCRHGR TLDEAIVHAN DF-NYSYFGI KTLERSYLMH IKDRLV-ERP QYLLMRCALG IHGENIDAVI ETYNYMSRHY FTHATPTLFS AATPKPQLSS CYLLTIKGDS IDGIFRTVAD CAAISKTAGG
 phycovirus  IIECIRSNAV VLDDVINYEK DL-EYDIFGY KTMEKSYLSR INGDIV-ERP QHMLMRVAIG IHCPDVDAAI ETYKLLSARV FTHATPTMYN AGTRNPQMSS CFLLKMKDDS IEGIFDTLKN CALISKNAGG
 herpesviru  IFKLSQEHAE ELDAALIHGN DH-TYSYFGF KTLERSYLLK GPKGPI-ERP QHMLMRCALQ IHKHDIPSVL ETYRLLSNKF MIHASPTLFN SGSSRPQLSS CFLLNLTDDS MAGIMETVRR CAMISKYAGG
  nudivirus  YFDLVQEHAD TLDSAIVDDR DFQSLTYFAY KTLERTYLIK LADGKICERV QHMYMRTALG IHGRDIESVI ETYNLLSNRY YVHSTPTLCH AATNRSNYAS CYLLELKEDS IGGVMDTLKD AATISKHCGG
   poxvirus                                                                                                                     MSS CFLLNMIDDS IEGIYDTLKR CALISKMAGG
       WSSV                                                                                                                     LSS CFLLGLQDDS IEGIYDTLKE AAIISKTAGG
  Consensus  .......... .......... .. ....... .......... ...... ... .......... .......... .......... .......... .......$sS C%LL...#DS IeGI.#Tlkr cA.!Sk.aGG

             261                                                                                                                                        390
       AmFV  VGLNISNVRA SGTPIRSTNG RSGGIIPMIR VFNNVARYVD QGGGKRPGAF TIYLEPWHAD IERFLLLSDK TGTEELIARD IYTAVWCCDL FMRRVERDET WSLMCPNLSP GLVDSWGDEF EKLYLEYERA
 baculoviru  IGFHVHNVRA KGSSVAGVGG ASNGLVPMLR VYNNTARYVD QGGNKRPGAF AVYLEPWHAD VLDFLDLKKN TGKEEVRARE LFYALWIPDL FMRRVENDSM WSLMCPMQSP GLADCYGAEF DALYQKYEEE
       SGHV  IGLNVHDIRA RGSPIDGTNG KSNGLVPMLR VFNNVARYVD QGGGKRMGSF AIYLEPWHAD IFEFLDLRKN HGIEEQRARD LFYALWIPDL FMRRVEQDGD WSLMCPHKCP CLSEVWGESF EHLYEHYERK
 phycovirus  IGVSIHNVRA QNSYIRGTGG YSNGLVPMLR VFDNTARYVD QGGGKRKGAF AMYLEPHHAD IFEFLELRKN TGKEELRARD LFYALWVSDH FMKRVEAGKN WSLFCPHECP GLSDVWGDDY VALYEKYESE
 herpesviru  IGLSMSNLRA SGSYIAGTNG QSNGLVPVLR IFNMVARLVD QGG-KRPGAF AIYMEPWHAD IFDFLDLRKN SGVDERRTRD LFTALWVPDL FMQRVRDDGD WSLMCPASCP GLDRVWGHEF NELYEQYEAT
  nudivirus  VGLHVHKLRC KNSTIHSTNG NASGLVPMLR MYNAMSRYVT QGGNKRPGAI AVYLEPWHAD VFDFIEMRKN SGFEEARARD LFYALWVPDL FMKRVNDGGY WSLMCPDSSP GLSDCWGEEF EAKYTAYEQE
   poxvirus  IGLSISNIRA SGSYISGTNG TSNGIIPMLR VYNNTARYID QGGNKRPGVM AIYLEPWHSD IMAFLDLKKN TGNEEHRTRD LFIALWIPDL FMKRVKDDGE WSLMCPDECP GLDDVWGDEF ERLYTQYERE
       WSSV  LGIHFHDLRA KGSPISSWSG THPGLMAFLQ IFNVSVKKVS QGGDKRRGAA AIYISDWHLD VKDFIDCRKN AGNEDLRTRD LFPAIWVSDL FMERVKAGKN WSLMCPHECP GLSDVHGEEF KALYEKYEAE
  Consensus  .Gl.is#.RA sgspI.stnG .s.Gl.pmlr !%Nn.ary!d QGG.KRpGa. a!YlepWHaD !..Fldlrkn tG.E#lraRD l%.AlW..DL FM.RV..d.. WSLMCP..cP GL.#vwGd#F e.LY..YE.e

             391                                                                                                                                        520
       AmFV  -GRFVRQVPA KKIWNTMVRM QLETGMPFLL HKDHINARSN QRHRGTIRGS NLCTEITLHT SAQEVAVCNL ASISLTPLTR DATREECKLC RTFDQDHLTN DFSIYGYRDD CTMCSGGFNF RELYHITRLV
 baculoviru  -GRYVRRVHA QTMWRAIIES QVETGTPYML YKDSCNRKSN QSNLGTIKCS NLCTEIVEYT SKDEVAVCNL ASIAVNKFVL QSGV-----Y ---------- ---------- --------DF EKLKKITKIV
       SGHV  -GRAVRTVKA RALWHAIIES QVETGTPYMM YKDACNRKSN QQNLGTIRCS NLCTEIVEYT APDEIAVCNL ASLALPKFVV SGTPDRPKHF ---------- ---------- --------DF EQLRRIVHVV
 phycovirus  -GKARKTIPA QKLWFAIMDS QIETGTPYML YKDSCNRKSN QQHLGTIQQS NLCTEIIEYT APDETAVCNL ASIALPAFVK DGS------F ---------- ---------- --------DH QQLYDVTYHA
 herpesviru  PGAVRRVVKA RYLWSQILVS QLETGSPYML YKDSANAKSN HQNLGTIRCS NLCTEIMEYT DSQEVAVCNL ASIALPEFVQ DGGYV----F ---------- ---------- --------NH EYLAHVTKVA
  nudivirus  -GRYVRRVDA RELWKAIVTA QVETGTPYIL YKDTVNRNSN QRHLGTIKGS NLCTEIVEYA SPDETAVCVL ASVNLPKFVE GKV------F ---------- ---------- --------NL NLLAKTVRLV
   poxvirus  -KRYKSIIKA RVVWKAIIES QIETGTPFIL YKDACNKKSN QQNLGTIKCS NLCTEIIQYA DANEVAVCNL ASVALNMFVI DGR------F ---------- ---------- --------DF LKLKDVVKVI
       WSSV  -GKGKEVVKA RALFDQINSA RIETGTPYVC FKDTINRKSN QENVGIIKSS NLCTEIVQYS DSEETAVCNL ASIAVNKFVK YSPIPSLRPY ---------- ---------- -------VDY REMKRVVKIM
  Consensus   gr..r.!kA r.lw..i... q.ETGtP%.l yKD..N.kSN Q.nlGtIk.S NLCTEI..y. ..#EvAVCNL AS!al..fv. d......... .......... .......... ........#f ..$..!vk..

             521                                                                                                                                        650
       AmFV  VRNLDRTIDV MHYPLVEAER ANKLHRPLGI GVQGLANVFA KLRIPWNSSK ASLLNRRIFE TLYRAALHES CDLARVHGMP YLTYALSPAE RSGMMQHDLF TEWANKFVRR NDNEAGVDAL KNVYDEKEEG
 baculoviru  TRNLNRTIDS NWYPLPEAKV SNLKNRPIGI GVQGMADAFV MMRMPYESVA AAELNKKIFE TIYYGALEAS CELAAIYG-P YETYEGSPAS R-GVLQYDMW ---------- ---------- -D--NVTPSD
       SGHV  TRNLNRIIDI NYYPVPEARR SNLRHRPIGI GVQGLADTFV LMGMPYESDE ARELNRRIFE TIYFAALETS CQLAKEEG-V YDSFLGSPAS I-GTLQQDMW ---------- ---------- -PV-DVDDKP
 phycovirus  TCNLDKVIDK NYYPIPEAER SNMRHRPIGL GVQGLADVFM MLKMAFDSPD ARVLNKEIFE TMYFAAMTAS CDLAKTKG-K YSTYEGSPLS K-GKFQFDLW ---------- ---------- ----NVTPSV
 herpesviru  TRNLNKIIDC NFYPLEECSK SNLRHRPIGI GVQGLADVFQ RMMMPFTSPE AKKLNREIFE TIYYAALQAS CELAKEHG-P YSSYQGSPVS K-GILQFDMW ---------- ---------- -NVKDEDLSG
  nudivirus  TRNLNKIIDN ALYPIPSAAK SNFKHRPIGI GVQGYADALA MMGIAYE--D SMQLNRDIFE TIYHAALTES CQLARVHG-V YESYHGSPTS R-GELHFDRY ---------- ---------- -SV----HSQ
   poxvirus  VRNLNKIIDI NYYPIPEAEI SNKRHRPIGI GVQGLADAFI LLNYPFDSLE AQDLNKKIFE TIYYGALEAS CELAEKEG-P YETYTGSYAS N-GILQYDLW ---------- ---------- -NV---VPSD
       WSSV  TRNLDKVIDV NFYAVDKTRI SNMKTRPMGL GVQGLADLFF KLRIPFESEE AALINKRIFE TIYYGALEAS CEIAKEKGET YELFEGSPLS K-GIFQFDMG ---------- ---------- -KENIKNRDI
  Consensus  tRNL#k.ID. n.Yp..eae. sN..hRPiGi GVQG$A#.F. .$ripf#S.e A..lNkrIFE TiYyaAL.aS C#lA...G.p Yet%.gSpas ..G..Q.D$. .......... .......... .nv.......

             651                                                                                                                                        780
       AmFV  AWLTAVCPEA WSWTELRNQI EEHGLRNSQR LAPMPTASTA QILGNVESIE PLTTNFFSRR VRSGEFAVVN QYLVDDLVRL GLWNEKMQQR IIRARGSVQN IERLPFRLRE VYRTVWEMPQ RLLVDLCAAR
 baculoviru  LW------D- --WSMLKSKI SKHGLRNSLL LAPMPTASTA QILGNNESFE PFTSNIYQRR VLSGEFQVVN RHLVKDLTAI GLWNDTIKNL IIHNNGSVQR IESIPLEIRQ LYKTVWEMSM RTLINMAADR
       SGHV  MW------N- --WDELRHNI EMNGLRNSLL VAPMPTASTA QILGNTESFE PITSNIYVRR VLSGEFQVIN RHLVQHLIDL GLWSESMRNR IIARRGSVQD IEEIPIEVRR LYKTVWEISG RRIIDMAADR
 phycovirus  RW------N- --WDTLRSNV KKHGTRNSLV TAPMPTASTA QILGNNECFE PFTSNLYSRR VLSGEFPVVN KYLVKDLSDR GLWGDSMKNY IIAANGSVQN IPGFPEDLKP IYKTVWEISM RTLIDMAADR
 herpesviru  RW------D- --WAELRRLI GEHGVRNSLL VAPMPTASTA QILGNNESIE PYTSNFYQRR VLSGDFQIVN PHLVKCLETR GLWNEDMRLQ LTIHRGSVQN IPGFPEDLKE IFKTVWELSQ RDIIDMAADR
  nudivirus  KC------D- --WSSLRKDI ARYGLRNSLL VAPMPTATTS QVFGNAESFE PFTSNMYQRR TQSGEFQLTN VHLVRDLERL NLWNDDMAQL IMYNNGSIQN ITIIPEELRR IYKTVWEIPT KTLIDMAADR
   poxvirus  LW------D- --WESLKDKI RSYGLRNSLL VAPMPTASTA QILGNNESVE PYTSNIYTRR VLSGEFQVVN PHLLRVLTER KLWNEEIKNR IMADGGSIQN -TNLPEDIKR VYKTIWEIPQ KTIIKMAADR
       WSSV  YFNSLPIHD- --WEQLRRDI MKYGVHNSMF VAPMPTASTA QILGNSESFE PLTSNMYNRN VLSGSFQVVN EYVIRELIKL GEWNSVTKQR IMASGGSIQT LPNIPKSTKE LFKTVWEINP RTTLDMAIQR
  Consensus  .w......#. ..W..Lr..I ..yGlrNSl. vAPMPTASTA QILGN.ES.E P.TsN.%.Rr VlSGeFq!!N .ylvr.L..l glWne.mk#r Ima..GS!Qn i...P..lke .%kT!WEipq rt.id$aadR

             781                                                                                                                                        910
       AmFV  QPFVDQSQSL NLYFPVPN-- ---FKRLTNA HFYGWRNGLK TGMYYL
 baculoviru  GAFIDQSQSF NVYMDLPS-- ---YGKLTSI HFYAWKMGLK TGMYYLRTKP AANAIQFTVD KTAA------ --F-AACNSC SS
       SGHV  GRYIDQSQSL NMHVAEPN-- ---YAKLSSM HFYAWRAGLK TGMYYLRTKA AANAIQFTVD SSSG------ --V-SSSASS TQDGPACTRE -CLSCSS
 phycovirus  GAFICQSQSF NVFMTQPT-- ---SAKLTSM HFYGWKSGLK TGAIAIYHIQ YTHYIS
 herpesviru  APFIDQSQSL NLHLGHEDDQ NIKLKKLTAM HMHGWGKGLK TGMYYLRTKA AADPVQFTVM PSTKRPCPDD GEK-RSRKSP KTTNIECTDD VCVMCSS
  nudivirus  GVYIDQSQSF NLYVAEPT-- ---YSRMTSI HFYAWNKGLK TGMYYLRTKS ATNAIPFTVD VNRCALLMSQ MKAGRVMASP FTKSLLVVEG SSGGVESTTT GSNTSNSTTS SSTSSSNPSS TPSSTGTKRN
   poxvirus  GAFIDQSQSM NIHIADPS-- ---YSKLTSM HFYGWSLGLK TGMYYL
       WSSV  GMFVDQAQSL NLFVEEPE-- ---LSKVRSM TMYAWEKGIK T-LYYL
  Consensus  g.%!DQsQSl Nl....P.       .skltsm hfYgW..GlK Tg$YYL.... .......... .......... ... ...... .......... .......

             911                                                                                                                   1027
       AmFV
```

```
baculoviru
      SGHV
phycovirus
herpesviru
 nudivirus  LSRTMRSKRS LHHITFMQQI AENPTKAPLK AKIDAANSVE NLSKRKEPSF ELVDSDAKKI CMDIDDKSKD DENNSQMCQL EPKPTEVNES IEPKESIELT GRNQMVCFDD VCTSCSC
  poxvirus
      WSSV
 Consensus
```

P74 (AmFV_79)

Go directly to Alignment

```
Multalin version 5.4.1
Copyright I.N.R.A. France 1989, 1991, 1994, 1996
Published research using this software should cite
Multiple sequence alignment with hierarchical clustering
F. CORPET, 1988, Nucl. Acids Res., 16 (22), 10881-10890
Symbol comparison table: blosum62
Gap weight: 12
Gap length weight: 2
Consensus levels: high=90% low=50%
Consensus symbols:
 ! is anyone of IV
 $ is anyone of LM
 % is anyone of FY
 # is anyone of NDQEBZ

 MSF:   1227    Check:   0          ..
 Name: bracovirus     Len:  1227  Check: 4564  Weight:  1.11
 Name: nudivirus      Len:  1227  Check:  612  Weight:  0.99
 Name: SGHV           Len:  1227  Check: 3976  Weight:  0.99
 Name: baculovirus    Len:  1227  Check: 8726  Weight:  1.01
 Name: WSSV           Len:  1227  Check: 7891  Weight:  0.95
 Name: AmFV           Len:  1227  Check: 3844  Weight:  0.95
 Name: Consensus      Len:  1227  Check: 2043  Weight:  0.00

//

            1                                                                                                                                130
bracovirus
 nudivirus  MSTVPTNLNP NPNPTTPTNP TTTTTTTTTY TQLDIANASK FSYHRKLLML LHKLNEKYPF LASHLKVNV- RAATDADYYI PPAMASEAIF VEAEITK--Q LCESISCNSS GVHGP-CKKT DAASYYRLGE
      SGHV                           MSRRFNPP TQLDYSYADL YKYERYKIQQ YNYFLCYRPE IMSHTYIEI- SYNGDLPNDN LPWYRKIRFV VRSR--K--K FCSQTSCQIN YPRGKMCQSD DKPRIFKTGD
baculoviru                               MAVL TAVDLTNASR YAGHMHRLEF INRWRERLPH ILIDYTLR-- PASSDDDYYV PPNLRNRALA VKLAFSR--R GCDSMSCFPF HETGVVSSQT PFA-YTQTSE
      WSSV
      AmFV                          M IIPDDAYPSW TNADARDANL YSIHRQRLWF SNYCVSIWPQ IFSWIVVNIY PASSEHDYRF EPQFANRAIL VTVDWDRSGR GCRNSKCYAT YPPGNRCTMS EAPIVFPSGN
 Consensus  .......... .......... .......... t..d...a.. .......... ........p. .......... .......... .p........ v......... .c....c... ...g...... ..........

            131                                                                                                                              260
bracovirus
 nudivirus  SENFEVQCNP ACFHLFE-TA VHTDDGTDKP HNVRYR---- -WSETSNTCN IVPFAATWME IPRYRSSELY A-PRLNDLDT GFDY------ ---------- -EPSTDT--- ----YHFNKY YCDVYYEAFD
      SGHV  HDL--EACQF SCYHLYEMTK TPSETKSDEK DDDYMRAPFL IYSYGQCACT IHSNGFFTLG ADDYARTDTH PKPRIDTIGT GFHYVDSGNF FDREDFSPED NQPYRDTEGN ESFRFQVNKY YCDDFQLKFD
baculoviru  TSV--AYAQP ACYHLDRAAA MREGAENEVQ SAEFTYTP-- -----NNQCV LVDSTSKMYF NSPYLRTEEH TIMGVDDV-P AFNV------ ---------- -RPDPDPLFP ERFKGEFNEA YCRRFGR--D
      WSSV                                            MATFTEQDHK NAFLYANEKL RQERIYRLKM SEPSVYAF-I DIKEIENGW- ---EKEFGLL VQPGQKLAPF RDISYDSSKL DCDAFSCIPS
      AmFV  KSNV-VACQP ACFARQRTRL LLKTADQEVS LTDQSGPSRS CPDCAYLNER VAFSTRRWRF GGSDGTRIDV FDSRTKKI-V GVQHERIGRT PVERDEHGSA KLKVDLFNVV WDEKYDNCIV VNDYVYRSVV
 Consensus  .......... .c........ .......... .......... .......... .......... .......... .......... .......... .......... ..p....... .......... .cd.......

            261                                                                                                                              390
```

```
 bracovirus
  nudivirus  PDTRTCYTKW YNKLANIIVG DAVIKLINAG LTAMSNGNGK T---LPPLEK DPPPAIDDKW TVSGWRKNID PNFKLPPIDV TVSDL----E TMNRVNHRSK RS-PNTI--P MADNVKI--- VRFDDAMMKS
       SGHV  G--QKCYESV GEKIFGFLVS STLYKACQYG VRYAATGVSN TD--VQKLDL PPPRFHVHHE TYDSWKNDID PDAFFIDPNV SLLDLGFKDE TMKHCIFTTQ YGYPGRLVEP KATGKNLTGN IVDYEALNRG
 baculoviru  LINGGCSFRW WESLIGFVLG DTIYVTFKML ANNIFSELRD FDYAAPSPLL PPRPAADSNA VLAQWRAVRD RAVDW----- ---------- -----DFEKQ FSEAPTL--- ---------- ---QQLGMDA
       WSSV  DILHSDNEKR V-GECNFAEH TSVSFPVKNP EGKTL----R HFTACGPGCY RRYKQRDPHT GLPVARGVLM QDHVDHETGN KMCEYLNQSL VMW---AAVP WIRPGDL--T EGYNTTHVPG FAFK--EDDE
       AmFV  EPLYRDPGVV C-KLTNFELG DDVEWGFRYA ENSSRVVPNY GFLVKHSSAY CRAFAKDL-T SEGECKQPWW QSALANTIAG DGIVHAIHSL VHYDPDCVEP WRTQENE--R AQTPQTFEAD TVYDLWRRDV
  Consensus  .......... ......f... .......... .......... .......... ......d... .......... .......... .......... .......... .......... .......... ..........

             391                                                                                                                                  520
 bracovirus                                                                                                                              MKDRSTT MSLKIQILTL
  nudivirus  KPKPTKPSTS TSTSTTKNQD IEKLLNLVKS LNDTSGADRE LKNALASQTG KQTKFESIIL SLLNELTSAD FAASVAVDVG VTQ--LASQL KTMTVKAIQK LVPLLQKKVA TMAIDTSELV LGASFKVAVR
       SGHV  RLYQFRYDVN SGRRLIDEYE IYDIYKYIRS NPTNTPDDRD YMQARQNLAE ILHGIISN-L GEVGGMMALG YLIDKGVQYS TNL--IKLST EYLEGKITPT LLHIVERELA SKALNPAIQM FSKAIASVAR
 baculoviru  NGVLMQLSYT AETGFTKTPI AYSARGAVRV ARESRAADRA MSD------- --DDLEAIVA SFLEEYA--- LVFGIATDIG FDM--LLTAF KAMLKKINTA LIPALKRMLV GTSQRVTVRL LGETYKAALV
       WSSV  RDSKRVKYEN VVISKAYCDF F----KQYYD ADSGSCYRSG WMKFVHLMFG QYFTNLSYNL A---NPKPYN LTGNTWSDVV SV---LTDNP IVDAGAAPS- ------RSEM DEIITKKKFN VFPSEQTSAR
       AmFV  NDKFVLPPPN VTLSDLGINV LLTGNRLYWN SAEGIISQFA LFRSVTNTPG TLSPNEPSTT TPNRRNRPLL LDSGVENERM KYQRYTTKPP KRRWGKRAAF INRLRRETRA NEAMRQHATN VLTEYRDLLR
  Consensus  .......... .......... .......... .......... .......... .......... .......... .......... .......... .....k.... .......... .......... .........r

             521                                                                                                                                  650
 bracovirus  LPEKWNERRI CETMNTSRHL TRVAKKLVEE KGLLSTPESK LGRTITHQML LKIENFYNDD EYSALMLGMK DFVSVQNNDG NRVHVQKRLV LSNLKELYQC FREIN--PTE KIGFSKFASL RPKHCVLARA
  nudivirus  DLVVPLTVRS LGTI--GVYL TKSLAMASTG VGVVLLITQL FSILLTFWDP L----GFNNM -YTPDYLHQL YENSKMALR- SQLKTNRPVL TFDYV-CSFL LTEEDL---F DASIESYTYT YRYLDALEVN
       SGHV  ISGGLIKTMD VFTTVAGILD LIDVGADFFN MGSVMDTGTV HQYSQMDIDV LRRSYGYGTV EYSPVTFMLT CEMLKLYEKW SEIPLATKRL RNEHTKYKYM IPAEAVTRYH EDDNNSYEWV SEYVFSLRTN
 baculoviru  HSMNRIAIKT LTTA--AKAL TRVAIKASSV VGIVLILFTL ADLVLALWDP F----GYNNM FPREFPDDLS RTFLTAYFET LDSNTSREII EFLPEFFADI VETDD----- DATFQSLFHL LDYVAALEVN
       WSSV  QKAENIIRSQ YGDGVEIDPS SVDALMQFVN REGVVGTEKK SDRLMRVADA VMD--AAMRL QVMGLDDSQS RRLL-LKNMI KMSRNNPEYA RHFSSSLKLI GVTL---AIK RSVFSKGASA KRKETAINNG
       AmFV  AKIERFRDRA AMEGREREQR ERHARFTRSN STTTSASTTL PASTNASDDP ATG--GDSGG DRDRTWDRRS HGSFDRRNLL RDADIDSKLG RLWRDEVRPL LDRVNNDATG RSIVGGYTSA KVVGHDTTDE
  Consensus  ........r. ..t......l tr.a...... .g.v...... ........d. l....gyn.. .y........ .......n.. .......... .......... .......... ...f....s. ......l...

             651                                                                                                                                  780
 bracovirus  SGTHTICVCT IHQNFNDCLD MIICETPSVQ CYLGGCNNCP EVDELSNILL TCFENQEIEN ITYKYWISKP RSSLETFIKP TEEFIENFCS E---LKVLLP HSFIAKQQAK FLKTLKETLK PNEFVIICDF
  nudivirus  SDGARINKGN VIDVGSIE-- --FEKILTRA KLFTVQDFEV YESHHKLRRQ AFDTINRYTG IAVGIGVVLI VLRVYLLGVL MLLLSVALVS V-SYANIAFN VWQWITDRIN PFKV
       SGHV  SNGLDINWSE EQSLPDDV-- --AQQYTRID KNLYYQGIDE YATYTQSFRK RVQ-FSRY-- VLVAVIIIFV IIAFVYIQVA VPAIFIAAMS T-FY--LVFS YFQ
 baculoviru  SDGQMLALDE SDEIKDFDEA TLVGQALASS SLYTRLEFMQ YTYRQNTLLA MNKNNNKLNG VIAGLFLTNT AVALAAFIAH KELTFFVYFA I-F---LMLA FYYLVKEPYE YFKTVDLLF
       WSSV  EQHRRSRWSP ETVTEEDALL FARENITEDP KHPAPFVDIL HSPDINSSIK SGSSSSIWND ILSRISSTRK LEEKASVFVK NLVVKVVRQF LDILEGKLFS DGYEWDDNIP LMIGVDQILR EVIKAASNMC
       AmFV  ELYPSSAVTD DE-EEELTSI YTRAGSVSYT LETNELAAQR YVESRLADDE QRKPKSATAT ATVTTGTTDT TDKTNTDDGS TATTSSPRLL VPSTARTIGW RSGEAEDP-D LKDLLEHIQE EIENGEASTL
  Consensus  s....i.... ......d... .......... .......... y......... .......... i......... .......... .........s .......... .......... ..k....... ..........

             781                                                                                                                                  910
 bracovirus  AENYAFVVQN AASGFHWNNN QATVFPVV-- IYYKINDELD HKSLVIISDC NNHDAVAVHV FIKLITDYVK SLPERCNKIY YFSDGAPQQF KNFKNFVNLH YHEDDFDIPA EWHFSATAHG KGPCDGIGGI
  nudivirus
       SGHV
 baculoviru
       WSSV  ARFASSALES SLVTGFIDSA SAITSRLAVQ LAARTFSVFL EESVIEFVVA ASL-----RL AIQAFADLAT LAASALTVIG IVIFVIQVLG LILDLALGLG WYDHIFSPED LKKQVLVFRR EFAKAGNVDV
       AmFV  LQDLGIAVGF EVADRLIRKS LKFAFTKISQ ILSRHTSGVI GKSLMKIGMR VGLSKMFGNV AARLTTKVMV MIGQAASIVG LIIDTISFVS LLFDIAELAG WDPGSFRNEQ DTHFYYDMSV YFAET--LTD
  Consensus  .......... .......... .......... .......... ..s....... .......... .......... .......... .......... .......... .....f.... .......... ..........

             911                                                                                                                                 1040
 bracovirus  IKRHAARASL QLAVDKQITT PIEFFE
  nudivirus
       SGHV
 baculoviru
       WSSV  GVAQPVTPEE IVAINVFLQT EENGEEK--K EEGARKSKID FL--QKYFHS TPLMGKKSKF VYIQEAAQEY LGGRTMNAFG Q-----RIIT AADDSDTTTT TQEGRRDDET VTKKMRSIIL ET---GQTLK
       AmFV  TQRGPLSPLE MMLIFTTDDT AADGNQKGNT NETAVNDNAD NTGTQEESGS TKTQHIDERL RYPKWFNKQF TACFPKNAFD VFNGVVNNFS GASNDPEHVV IQDSAEIWGL VTCMDYLGSL TTNSFGQRIV
  Consensus  .......... .........t .......... .......... .......... .......... .......... .......... .......... .......... .......... .......... ..........

             1041                                                                                                                                1170
 bracovirus
  nudivirus
       SGHV
 baculoviru
       WSSV  DYSSAVNYNA SRLDYVGEEW VRNTALKEET RSNTTSDNLF KKT------- ---VSLASMA GAFLVLG-IG VLVASHITLL RFTNIGLAFA F---AGLLAF IALMSISYIN MNAMGVVNSD AIYRSTALVG
       AmFV  SDPVNVRLRD EDLSNLVTEI EYRDLLTIST RSSVDSTSFN HRVNASNRIQ VIGLAVAGVA GVGTVVSLIG SNLFNTYTGT RPPWYLILFD FNVISILIVF CALVGFLYAS LSFVPVPVTT IASNDSSGTT
  Consensus  .......... .......... .......... .......... .......... .......... .......... .......... .......... .......... .......... .......... ..........

             1171                                                    1227
 bracovirus
  nudivirus
       SGHV
```

```
baculoviru
      WSSV  DIKTDPRRVG MVQRHVGVGA KYNMITDFVS PMLDEIESD
      AmFV  TTGGTTTRAL ATTTGTGGGT GTTGTTDKND NNSSTSTEHQ TLHYYADTLR NFATTLS
 Consensus  .......... .......... .......... .......... .......... .......
```
