# Supplementary material for: The Apis mellifera Filamentous Virus Genome
Source: Viruses. 2015 Jul 9;7(7):3798–815. doi: 10.3390/v7072798 (PMC4517127; doi:10.3390/v7072798)
Supplement: Supplementary file 1 [file viruses-07-02798-s001.zip › FigureS1.pdf]

Figure S1. Alignments of sequences obtained from Swiss and US AmFV isolates. The following genes were used for designing diagnostic PCR assays: AmFV_112 (*Bro* gene), AmFV_28 (thymidylate synthase gene) and AmFV_221 (ribonucleotide reductase gene). Primers positions are underlined in bold characters.

1. AmFV_112 (Bro gene)

```
CLUSTAL 2.1 Multiple Sequence Alignments


Sequence type explicitly set to DNA
Sequence format is Pearson
Sequence 1: AmFV_112    1497 bp
Sequence 2: US          1497 bp
Start of Pairwise alignments
Aligning...



Sequences (1:2) Aligned. Score: 98.33
Guide tree file created:   [clustalw.dnd]

There are 1 groups
Start of Multiple Alignment

Aligning...
Group 1: Sequences:   2      Score:28110
Alignment Score 10157



CLUSTAL 2.1 multiple sequence alignment


AmFV_112_CH_      ATGGATTCAAAGGTTTATACCTATGAAGATCAAGAGGTTACTGTACTTAGTTGTTTAGAC
US_BRO            ATGGAGTCAAAGGTTTATACCTATGAAGATCAAGAGGTTACTGTACTTAGTTGTTTAGAC
                  ***** ******************************************************

AmFV_112_CH_      GAAAACAATGTACGTTTTTATTATCTAATAACGTTTCTTGCTATTTTTGGTCTAAAAAAC
US_BRO            GAAAACAATGTACGTTTTTATTATCTAATAACTTTTCTTGCTATTTTTGGTCTAAAAAAC
                  ******************************** ***************************

AmFV_112_CH_      CGACGTTCGTACATAAGAACACGCTTACAAAATCTGCAAAAAACCGCAAAGATATTTCTT
US_BRO            CAACGTTCGTACATAAGAACACGCTTACAAAATCTGCAAAAAAATGCAAAGATTTTCCTT
                  * *****************************************  ******** ** ***

AmFV_112_CH_      CGATATCTATCCGAATTTAATGTATCGAGCGTCTTACCAGAGAATTCGGTTTTTGTGAGT
US_BRO            CGATATCTATCTGAATTCAATGTATCGAGTGTCTTACCAGAGAATTCGGTTTTTGTGAGT
                  *********** ***** *********** ******************************

AmFV_112_CH_      GCTGCTGGATTAAAAGAAATTATATCAAAGTTACAACATGAGAAAAAACATTTTTGGATT
US_BRO            GCTGCCGGATTAAGAGAAATTATATCAAAGTTACAACATGAGAAAAAAAATTATTTGATT
                  ***** ******* ********************************** *** ** ****

AmFV_112_CH_      ACCTTGTTGAAGAGAATTTTTCGTGATTATAAAGAAAGTAGTTGGTCAAACAAAAACGAC
US_BRO            ACCTTTTTGAAAAGGATTTTTCGTGATTATAAAGAAAGTAGTTGGTCAAACAAAAACGAC
                  ***** ***** ** *********************************************

AmFV_112_CH_      ATTTTAATGAAAAAGGTAACTTTTGAAGGATATTCTTTTGACTTGGTGAAGATAATTGGT
US_BRO            ATTTTAATGAAAAAAGTAACTTTTGAAGGATATTCTTTTGACTTGGTGAAGATAATTGGT
                  ************** *********************************************

AmFV_112_CH_      GATGACGATGATTATTTTTACGCTAAACCACTCGAAAAAATTTTGAATTACAAGAAGCTA
US_BRO            GATGACGATGATTATTTTTACGCTAAACCACTGGAAAAAATTTTGAATTACAAGAAGCTA
                  ******************************** ***************************
```

```
AmFV_112_CH_      CATAAAGCTATACGAAAGTACGTTTCGCCGAACAATTGTCGTCCATTTAGTTCGTTCGAC
US_BRO            CATAAAGCTATACAAAAGTACGTTTCGCCGAACAATTGTCGTCCATTTAGTTCGTTCGAC
                  ************* **********************************************

AmFV_112_CH_      ATAGGGGTTATATACACAGGAAAAACAACATCGTTGCCATATCTCAAACCGAATGCAAAC
US_BRO            ATAGGGGTTATATACACAGGAAAAACGACATCGTTGCCATATCTCAAACCGAATGCAAAC
                  ************************** *********************************

AmFV_112_CH_      TTTATTAACACCGCAGGCTTCAACGAATTAATTTTGCATTCGACCCAACCTTTTGCGAAG
US_BRO            TTTATTAACACCGCAGGCTTCAACGAATTAATTTTGCATTCGACCCAACCTTTTGCGAAG
                  ************************************************************

AmFV_112_CH_      CGTTTCAAGGAATGGCTGATCAACGTTATGGATCAATTGCGAGTGGACGGCGAGTACGTG
US_BRO            CGTTTCAAGGAATGGCTGATCAACGTTATGGATCAATTGCGAGTGGACGGCGAGTACGTG
                  ************************************************************

AmFV_112_CH_      GTTACCCGAGACGCCCCATTGGACATCCAGCAAGACTTGGCCACCATGAACAACATCTTT
US_BRO            GTTACCCGAGACGCCCCATTGGACATCCAGCAAGACTTGGCCACCATGAACAACATCTTT
                  ************************************************************

AmFV_112_CH_      GGACAGACACCTCGGACACCTCGATTAGCTGTTCAACCTCTTCAGACGAAACCTCTGGAT
US_BRO            GGACAGACACCTCGGACACCTCGATTAGCTGTTCAACCTCTTCAGACGAAACCTCTGGAT
                  ************************************************************

AmFV_112_CH_      CAGTTGACTCTATCGGAGTGTCACGACTTGATAATGTTCTTACAAAGAGAAAACAACTTA
US_BRO            CAGTTGACTCTATCGGAGTGTCACGACTTGATAATGTTCTTACAAAGAGAAAACAACTTA
                  ************************************************************

AmFV_112_CH_      TACAAACGGAACATACAGAGATATCAAGTCCAACTACGCCAACGTGTCACTCATCCGGCA
US_BRO            TACAAACGGAACATACAGAGATATCAAGTCCAACTACGCCAACGTGTCACTCATCCGGCA
                  ************************************************************

AmFV_112_CH_      GACTACAGTCTCTTCATGTTGCTGTCGATGAGTGTTGTCCGATCCGTCATTCGGTTCTCC
US_BRO            GACTACAGTCTCTTCATGTTGCTGTCGATGAGTGTTGTCCGATCCGTCATTCGGTTCTCC
                  ************************************************************

AmFV_112_CH_      ATCTCGAGAGGAAAGGACAAGTATATTCGTAGTATCGAGGCAAAACGTAGGAAATTTGTC
US_BRO            ATCTCGAGAGGAAAGGACAAGTATATTCGTAGTATCGAGGCAAAACGTAGGAAATTTGTC
                  ************************************************************

AmFV_112_CH_      GCCGCTTGCAAGCAAGACCGGTCTCTGCTGTCTTCCCCGCAAGCCGCGCGGTATCTGCAG
US_BRO            GCCGCTTGCAAGCAAGACCGGTCTCTGCTGTCTTCCCCGCAAGCCGCGCGGTATCTGCAG
                  ************************************************************

AmFV_112_CH_      ATTACCATCAAAAAGTACAGAACCGCCAACGCGGCGATCTCGTGGATGCGGATTCGTAAA
US_BRO            ATTACCATCAAAAAGTACAGAACCGCCAACGCGGCGATCTCGTGGATGCGGATTCGTAAA
                  ************************************************************

AmFV_112_CH_      GAGTATATAAACTTTTTGGACGACGTGGACTTTACGAATCGTTGCAAGACCGTTTTTTCG
US_BRO            GAGTATATGAACTTTTTGGACGACGTGGACTTTACGAATCGTTGCAAGACCGTTTTTTCG
                  ******** ***************************************************

AmFV_112_CH_      CCACGAGGTTTACGGCCCGCATCGATGGATCCCCTCTTCTCCGATCGGCTGGACGCACAT
US_BRO            CCACGAGGTTTACGGCCCGCATCGATGGATCCCCTCTTCTCCGATCGGCTGGACGCACAT
                  ************************************************************

AmFV_112_CH_      GACGACCGTGACTCTTTAGAGTGGCTGGACCCCAAAATAGCCGACTTCAAGGGCACGGTC
US_BRO            GACGACCGTGACTCTTTAGAGTGGCTGGACCCCAAAATAGCCGACTTCAAGGGCACGGTC
                  ************************************************************

AmFV_112_CH_      GAGCGTATGTGCGACAGAGTGTTCGAAAGGCTCGATCGGGAGACCAACTTGACGACTAAC
US_BRO            GAGCGTATGTGCGACAGAGTGTTCGAAAGGCTCGAGCGGGAGACCAACTTGACGACTAAC
                  *********************************** ************************

AmFV_112_CH_      TCGGCAAGAATAGAGGACGATCCGAGCAAGCCTGGACCGTCTAATGCGGTTGAGTAA
US_BRO            TCGGCAAGAATAGAGGACGATCCGAGCAAGCCTGGGCCGTCTAATGCGGTTGAGTAA
                  *********************************** *********************
```

2. AmFV_28 (thymidylate synthase gene)

```
CLUSTAL 2.1 Multiple Sequence Alignments


Sequence type explicitly set to DNA
Sequence format is Pearson
Sequence 1: AmFV_28    1833 bp
Sequence 2: US_TS     1833 bp
Start of Pairwise alignments
Aligning...



Sequences (1:2) Aligned. Score: 98.9634
Guide tree file created:   [clustalw.dnd]

There are 1 groups
Start of Multiple Alignment

Aligning...
Group 1: Sequences:   2      Score:34599
Alignment Score 13792

CLUSTAL-Alignment file created  [clustalw.aln]
```

```
clustalw.aln

CLUSTAL 2.1 multiple sequence alignment


AmFV_28         ATGAGTGCATCGAATCCTATTGAGATCAACTCTATCACTCAAACGAACGGGAATGCGGAA
US_TS           ATGAGTGCATCGAATCCTATTGAGATCAACTCGATCACTCAAACGAACGGGAATGCGGAA
                ******************************** ***************************

AmFV_28         CATCTTATTGATGACACTTACGGCCGGAACGAGACGCCTAACACCGGCGACACAGCACAA
US_TS           CATCTTATTGATGACACTTGCGGCCGGAACGAGACGCCTAACACCGGCGACACAGCACAA
                ******************* ****************************************

AmFV_28         CTCGTCGCATCGCCAGACACGCCAAAAACGACAGAAACTCCAGAAGTAGAAACGGCAACA
US_TS           CTCGTCGCATCGCCAGACACGCCAAAAACGACAGAAACTCCAGAAGTAGAAACGGCAACA
                ************************************************************

AmFV_28         ACGCCCGCCCAAATCAACACAGCCATCAATCGACCAACGAACGTAAGCCCTCAACATCCG
US_TS           ACGCCCGTCCAAATCAACACAGCCATCAATCGACCAACGAACGTAAGCCCTCAACATCCG
                ******* ****************************************************

AmFV_28         CCAAAACCGCCACGAAAGTCGATCGTGAATCCTCCGACTACTCCAAACGAAACACAAAAC
US_TS           CCAAAACCGCCACGAAAGTCGATCGTGAATCCTCCGACTACTCCAAACAAAACACAAAAC
                ************************************************ ***********

AmFV_28         GACCAACTAAGAACCTCACGCACCGACCAACCAAAACGCCAACCAAACCGCCAATTAAGC
US_TS           GACCAACTAAGAACCTCACACACCGACCAACCAAACCGCCAACCAAACCGCCAATTAAGC
                ******************* *************** ************************

AmFV_28         AAATTAAGCAACCCGCAGAGAGGTCCAAACTCCCCAAACCATCCACCAAGCGACTTTGTC
US_TS           AAATTAAGCAACCCGCAGAGAGGTCCAAACTCCCCAAACCGTCCACCAAGCGACTTTGTC
                **************************************** *******************

AmFV_28         AGAAACTCCTCCTCGAACAACTCGAACTGCTCACCCACAAACAACTCTAAACGCGGGGTC
US_TS           AGAAACTCCTCCTCGAACAACTCGAACTGCTCACCCACAAACAACTCTAAACGCGAGGTC
                ******************************************************* ****

AmFV_28         ACCACCAACTGGAACCACGCATTACCCTACGCTCCAGGCCACACACACAACAGAACAACG
US_TS           ACCACCAACTGGAACCACGCATTACCCTACGCTCCAGGTCACACACACAACAGAACAACG
```

************************************** *********************

AmFV_28 AACCACTCGTTGAACAACAAGTGGGACGAGTGGAACCGCGAACTCAGTCGAATCGAGAGA
US_TS AACCACTCGTTGAACAACAAGTGGGACGAGTGGAACCGCGAACTCAGTCGAATCGAGAGA
****

AmFV_28 CGCGTACAAACGATCAACGAGACCTTCAAAAACATTGGTGAGATGAGCACGGAACCAAGT
US_TS CGCGTACAAACGATCAACGAGACCTTCAAGAACATTGGTGAGATGAGCACGGAACCAAGT
***************************** ******************************

AmFV_28 GAAACTGAAAACCCCAATCAAAGTACCAGCGTAGCGAGCATCAACAATCTTGTCACTGAC
US_TS GAAACTAAAAACCCCAATCAAAGTACCAGCGTAGCGAGCATCAACAATCTTGTCACTGAC
****** ***

AmFV_28 ATCGACAGCGACATTATGGGCAACACATCCATCGACGCAACCGAAAAGGCGAGACTCCTC
US_TS ATCGACAGCGACATTATGGGCAACACATCCATCGACGCAACCGAAAAGGCAAGACTCCTC
** *********

AmFV_28 GCGATGATCGCCTCCACACCGGGACTGGAGAACGACGAGCCGGACAACGTCAACGTGAAC
US_TS GCGATGATCGCCTCCACACCGGGACTGGAGAACGACGAGCCGGACAACGTCAACGTGGAC
*** **

AmFV_28 GAGGTGAAAAACCTTCCCGATGTTCGATCCGACAACCGATACCACAACGATGCTCAAACC
US_TS GAGGTGAAAAACCTTCCCGATGTTCGATCCGACAACCGATACCACAACGATGCTAAAACC
** *****

AmFV_28 GATACTATCGACCGATTCGGTCTGACCGACCCGCAGTTCGACGACACATGGAACGAC**CGC**
US_TS GATACTATCGACCGATTCGGTCTGACCGACCCGCAGTTCGACGACACATGGAACGACCGC
****

AmFV_28 **ATGTACCAACAACTCGTAC**GAAAAGTACTGAAACGAGGCGAACAACGGGACGACCGCACG
US_TS ATGTACCAACAACTCGTACGAAAAGTACTGAAACGAGGCGAACAACGGGACGACCGCACG
****

AmFV_28 GGCACGGGAACTCGCGCCCTGTTCGGCGAGCTGCTCGAATTTGATCTTCGAGGTAATCGG
US_TS GGCACGGGAACTCGCGCCCTGTTCGGCGAGCTGCTCGAATTTGATCTTCGAGGTAATCGG
****

AmFV_28 CTGCCATTGCTCAACTGCCGCCGCCTCTCGTTCAAGTCCATTCTCGAGGAACTGCTGTGG
US_TS CTGCCATTGCTCAACTGCCGCCGCCTCTCGTTCAAGTCCATTCTCGAGGAACTGCTGTGG
****

AmFV_28 TTCTTGCGCGGGGAGACCGACAGCAAAATACTGGAACGCCGAGGTGTACATATCTGGACC
US_TS TTCTTGCGCGGGGAGACCGACAGCAAAATACTGGAACGCCGAGGTGTACATATCTGGACC
****

AmFV_28 GAACATGCGACCCGAGACGTCCTGACGAAAAGAGGCTTTCCATCGCGGCGGGAAGGCGAC
US_TS GAACATGCGACCCGAGACGTACTGACGAAAAGAGGCTTTCCATCGCGGCGGGAAGGCGAC
******************** ***************************************

AmFV_28 ATCGGACCGCTCTACGGATTCCAATGGCGGTTCGCCGG**ACTGCGCTACACCAACTGTG**AA
US_TS ATCGGACCGCTCTACGGATTCCAATGGCGGTTCGCCGGACTGCGCTACACCAACTGTGAA
****

AmFV_28 GCCAACTATGCGGCCCAAAAACGAACGAGAGGGTATGACCAAATCGCTTACATTTTAGAC
US_TS GCCAACTATGCGGCCCAAAAACGAACGAGAGGGTACGACCAAATCGCTTACATTTTAGAC
*********************************** ************************

AmFV_28 GAACTGACTGCTCGTCCACACAGTCGTCGTATCTTGCTCAACTCGTGGAATGTAGCCGAC
US_TS GAACTGACTGCTCGTCCACACAGTCGTCGTATCTTGCTCAACTCCTGGAATGTAGCCGAC
*** ****************

AmFV_28 ATCGCCGAAATGGTGTTGCCGCCTTGTCACGTTATGTTTCAACTCTTTGCCGACAATGCT
US_TS ATCGCCGAAATGGTGTTGCCGCCTTGTCACGTCATGTTTCAACTCTTTGCCGACAATGCT
******************************** ***************************

AmFV_28 AACGAACTGTCAGGAATGCTGTACTCGCGCAGCGCAGACATCGGTCTTGGTTTACCGTTC
US_TS AACGAACTGTCAGGAATGCTGTACTCGCGCAGCGCGGACATCGGTCTTGGTTTACCGTTC
*********************************** ************************

```
AmFV_28         AATATTGCGTCCTACGCCATCTTGCTACACGCCATAGCCAAAAAAGTGGGTCGAACAGCC
US_TS           AATATTGCGTCCTACGCCATCTTGCTACACGCCATAGCCAAAAAAGTGGGTCGAACAGCC
                ************************************************************

AmFV_28         AAAACGTTGAAGGTGATCATCGGTGATGCCCACATCTACAACGACCACATCGACGCAATG
US_TS           AAAACGTTGAAGGTGATCATCGGTGATGCCCACATCTACAACGACCACATCGACGCAATG
                ************************************************************

AmFV_28         ACCGAGATTGCGGCTCGCGAACCACGACCATCACCACGCGTTTACATCGACAGCAGGTCG
US_TS           ACCGAGATTGCGGCTCGCGAACCACGACCATCACCACGCGTTTACATCGACAGCAGGTCG
                ************************************************************

AmFV_28         TTCGACAAACCGCTCCACGAATACTCGACCGACTTTTTCCACCTGTTCAACTACCACCCA
US_TS           TTCGACAAACCGCTCCACGAATACTCGACCGACTTTTTCCACCTGTTCAACTACCACCCA
                ************************************************************

AmFV_28         CACGAACCCGTTCGTATGCGCGTGTCCGTATAG
US_TS           CACGAACCCGTTCGTATGCGCGTGTCCGTATAG
                *********************************
```

3. AmFV_221 (ribonucleotide reductase gene)

```
CLUSTAL 2.1 Multiple Sequence Alignments


Sequence type explicitly set to DNA
Sequence format is Pearson
Sequence 1: AmFV_221    2649 bp
Sequence 2: US_RR       2634 bp
Start of Pairwise alignments
Aligning...



Sequences (1:2) Aligned. Score: 98.4055
Guide tree file created:   [clustalw.dnd]

There are 1 groups
Start of Multiple Alignment

Aligning...
Group 1: Sequences:   2      Score:49515
Alignment Score 19334

CLUSTAL-Alignment file created  [clustalw.aln]
```

---

clustalw.aln

```
CLUSTAL 2.1 multiple sequence alignment


AmFV_221        ATGAGCTTGCCCGCGATAATTGATCAGAGTATTGCGGGCGCTCAGAGCATTCGAGGTGAA
US_RR           ATGAGCTTGCCCGCGATAATTGATCAAAGTATTGCGGGTGTTCAGAGCATTCGAGGTGAA
                ************************** *********** * *******************

AmFV_221        CAAACGACGAATCTTGACAGCGACATTAAGGAAGCTTCCTCAGACTCGCAAGACACCGAT
US_RR           CAAACGACGAATCTTGACAGCGACATTAAGGAAGATTCCTCAGACTCGCAAGACACCGAT
                ********************************** *************************

AmFV_221        TCCACAGTCGTTCCCATCCTACCCGCACCACTTGGCTTTGCCGACGAGTCCGAGATAGCA
US_RR           TCCACAGTCGTTCCCATCCTACCCGCACCACTTGGCTTTGCCGACGAGTCCGAGATAGCA
                ************************************************************

AmFV_221        GAACCGACTATCGAGCCAACTACCACACCGACCGCCGATCTTACCACCGTTGAGCCTACC
US_RR           GAACCGACTATCGAGCCAACTACCACACCGACCGCCGAGCTTACCACCGTTGAGCCTACC
                ************************************** *********************

AmFV_221        GCTGAGCTCACCGTTGAGCCTACCGCTGAGCTTACCGCTGAGCTTACCGCTGATCCGACC
US_RR           GCTGAGCTCACCGTTGAGCCTACCGCTGAGCTTACCG------------CTGATCCGACC
                *************************************            ***********

AmFV_221        GGAGCCGAACTCGAACCGATCGTGGATCTACCCAAAGTCCCGATCGAAGAACCAGCAGTG
US_RR           AGAGCCGAACTCGAACCGATCGTGGATCTACCCAAAATCCCGATCGAAGAACCAGCAGTG
                 *********************************** ***********************

AmFV_221        GTAGTGTGCGACGACACATACACAAACACGTACACCGAAACCAACGATTCGACGGATGAC
US_RR           GTAGTGTGCGACGACACATACACCAACACGTACACCGAAACCAACGATTCGACGGATGAC
                *********************** ************************************

AmFV_221        GATTTTCCCGATTTTGCGGACTTTCGTGGCGCTAGTATCGTCGATCGTTCCGAATCTCCC
US_RR           GATTTTCCCGATTTTGCGGACTTTCGTGGCGCTAGTATCGTCGATCGTTCCGAATCTCCC
                ************************************************************

AmFV_221        CGACCTGGGGGAACACCGCCGCCGGCCTATATAGAGCGCTCGTCCTCACCAGTGTACAGA
```

US_RR CGACCTGGGGGAACACCGCCGCCGGCCTATATAGAGCGCTCGTCCTCACCGGTGTACAGA
** *********

AmFV_221 ACCCTACCTTCTGTGTATACGGACGATTCGACCAACGACGTTTCGATCTACGAAGGAATA
US_RR ATCCTACCTTCTGTGTATACGGACGATTCGACCAACGACGTTTCGATCTACGAAGGAATA
* **

AmFV_221 ACACGTTTCGGTGAATCGATCGTCTCGGTTGACACAAGCTCCACCGATCCGACAAAACCG
US_RR ACACGTTTCGGTGAATCGATCGTCTCGGTTGACACAAGCTCCACCGATCCGACAAAACCG
****

AmFV_221 CTCAACTTTGCCGAACTGTGGCGCGACAGCATACATACACCACTGTTACCTCGTCTGATC
US_RR CTCAACTTTGCCGAACTGTGGCGCGACAGCATACATACACCGCTGTTACCTCGTCTGATC
*** ******************

AmFV_221 CGGAATCAACGATTTGCAACACCGACTTTTACAACGTTTAAACCTATGGAGGCACAACGT
US_RR CGGAATCAACGATTTGCAACACCGACTTTTACAACGTTTAAACCTATGGAGGCACAACGT
****

AmFV_221 CCCTTACGCAAATCACCACAAATAAATTTACTCGAATCGATTGCAAAAGACTCGTCGGAC
US_RR CCCTTACGCAAATCACCACAAATAAATTTACTCGAATCGATTGCAAAAGACTCGTCGGAC
****

AmFV_221 GAACACTCTAGCGACGAGAGTGAGATCGTCTCACCGACCATGGTGTCTCGACCCGTTCGT
US_RR GAACACTCTAGCGACGAGAGTGAGATCGTCTCACCGACCATGGTGTCTCGACCCGTTCGT
****

AmFV_221 CACGTTCGCTTCAACTTGAAAGACGACGACTCCGAGTCGGAGCATTCTACTGAGATGGTC
US_RR CACGTTCGCTTCAACTTGAAAGACGACGACTCCGAGTCGGAGCATTCTACTGAAATGGTC
*** ******

AmFV_221 GAGATCTCGGTAAAGACCGAGAGAGAAGAGTCTGCTGACGACTCGGTGGCGGAATCGATC
US_RR GAGATCTCGGTAAAGACCGAGAGAGAAGAGTCTGCTGACGACTCGGCGGCGGAATCGAAC
** *********** *

AmFV_221 GACTCCGAAGAAAACGTACCGTGGCGACAGGAAGGGAGCACGCTGGTCTTGCAAATACCG
US_RR GACTCCGAAGAAAACGTACCGTGGCGACAGGAAGGGAGCACGTTGGTCTTGCAAATACCG
** *****************

AmFV_221 GACCACATTATGGCTCGTCTCTACCGACCGAAGAAACAAAAACAGAAACAAAAACAGAAG
US_RR GACCACATTATGGCTCGTCTCTACCGACCGAAGAAACAAAAACAGAAACAAAAACAGAAG
****

AmFV_221 CAAAACCCGCAGAACAAGCTAAACCAGCAGAACAAGCCAAACCAGCAGAACAAGCTTGGT
US_RR CAAAACCCGCAGAACAAGCTAAACCAGCAGAACAAGCTAAACCAGCAGAACAAGCTTGGT
************************************ ***********************

AmFV_221 CAGCAGAACAGACCTGTACCACAGTGCGATTCGAGTGATCAAAACGATCGAAATGATTCA
US_RR CAGCAGAACAGACCCGTACCACAGTGCGATTCGAGTGATCAAAACGATCAAAATGATTCA
************** ********************************** **********

AmFV_221 ACGGCTTTAGAGGACAAAGACGAGTCGAAACCGGGCTCGATGAGCTCGACAAGCTCACAA
US_RR ACGGCTTTAGAGGACAAAGACGAGTCGAAACCGTGCTCGA---GCTCGACAAGCTCACAA
********************************* ******   *****************

AmFV_221 AAATCTACCGTGCGTACTCGAGCCACGTGGAGCAAACCCGAACGGCCTATTGTGCTGTCA
US_RR AAATCTACCGTGCGTACTCGAGCCACATGGAGCAAACCCGAACGGCCTATTGTGCTCTCA
************************** ***************************** ***

AmFV_221 GTGCCGCCGCCTGAGCCACCCATTGACTACTCTGATAGTAGCGACGACGATGACGATGTA
US_RR GTGCCGCCGCCTGAGCCACCCATTGACTACTCTGATAGTAGCGACGACGATGACGATGTA
****

AmFV_221 GTGGTGTTTGACCACAATTCGAAGAGCGGAACTGCTGATCG**AGATCGCCCGCTTTGTCGC**
US_RR GTGGTGTTTGACCACAATTCGAAGAGCGGAACTGCTGATCGAGATCGCCCGCTTTGTCGC
****

AmFV_221 **C**GATCCTGCCCCGACGTTCTTCGCAAACACGATCACGAGTCGAGCAACTCTGAATCGTCT
US_RR CGATCCTGCCCCGACGTTCTTCGCAAACACGATCACGAGTCGAGCGACTCTGAATCGTCT
*** **************

AmFV_221 CACGTTAATCGCATACCCGAGCGCGAGATCGAGAAGGTTACGCGGTTTTGCGATCGTGTT
US_RR CACGTTAATCGCATACCCGAGCGCGAGATCGAGAAGGTTACGCGGTACTGCGATCGTGTT
** ************

AmFV_221 CCTGAGCGAACCTCTTTTGAAATGGAACCGTCGCCGCTAGTGGAACCGCTGCTCGACGCC
US_RR CCTGAGCGAACCTCTTTTGAAATGGAACCGTCGCCGCTAGTGGAACCGCTGCTCGACGCC
****

AmFV_221 ACTAAAGATGAGGAGCGGTTCGTCTTGTTCCCCGTTCAGCATCCCGACATCTACGACATG
US_RR ACTAAAGATGAGGAGCGGTTCGTCTTGTTCCCCGTTCAGCATCCCGACATCTACGACATG
****

AmFV_221 TACAAACGACAAGTGGCCCAGTTTTGGACCATGGAGGAGATTGATCTCTCCAAGGACTTG
US_RR TACAAACGACAAGTGGCCCAGTTTTGGACCATGGAGGAGATTGATCTCTCCAAGGACTTG
****

AmFV_221 GCCGATTGGAACGAACGACTATCTAGCCATGAACGCGATTTCTTCAAATGCATCTTGGCC
US_RR GCCGATTGGAACGAACGACTATCTAGCCATGAACGCGACTTCTTCAAATGCATCTTGGCC
************************************* **********************

AmFV_221 TTCTTTGCACCGAGCGACGGTATCGTCGGTGAGAATCTGGTGACGCGTTTCTACGAAATG
US_RR TTCTTTGCACCAAGCGACGGTATCGTCGGTGAGAATCTGGTGACGCGTTTCTACGAGATG
*********** ** ***

AmFV_221 GT<u>**AGTGTACACCGAGGCCCGCT**</u>ACTTCTACGCGTTCCAAGCCGCAATGGAGAACATTCAC
US_RR GTAGTGTACACCGAGGCCCGCTACTTCTACGCGTTCCAAGCCGCAATGGAGAACATTCAC
****

AmFV_221 GCCGAGGTCTACAGTCAGTTGATCAAGACACTAATTCCGAACGAAGACGAACAGCGGTCC
US_RR GCCGAGGTCTACAGTCAGTTGATCAAGACACTAATTCCGAACGAAGACGAACAGCGGTCC
****

AmFV_221 ATCTTCTCATCGTTCATGACCACGCCAGGCATCACTGAAAAGACTCGCTGGGCAAAACAG
US_RR ATCTTCTCATCGTTCATGACCACGCCAGGCATCACTGAAAAGACTCGCTGGGCAAAACAG
****

AmFV_221 TGGCTCGAACGAGAAAATCTCAGCTTCGTCGAGCGACTAGTCGCTTTTGCTGTGGTCGAA
US_RR TGGCTCGAACGAGAAAATCTCAGCTTCGTCGAGCGACTAGTCGCTTTTGCTGTGGTCGAA
****

AmFV_221 GGACTGTTGTTTAGCGGCAGCTTTGCCTCCATCTTCTGGTTGAAAAAGCGTGGACTTATG
US_RR GGACTGTTGTTTAGCGGCAGCTTTGCCTCCATCTTCTGGTTGAAAAAGCGTGGACTTATG
****

AmFV_221 CACGGTCTGACCTTTAGTAACGAACTGATCAGTCGGGACGAGGGTTTGCATTGCGACTTC
US_RR CACGGTCTGACCTTTAGTAACGAACTGATCAGTCGGGACGAGGGTTTGCATTGCGACTTC
****

AmFV_221 GCCTGTCTTCTCTACACCAAACACATCGTGCACAAGCTGCCCAACAATGTGATCTACAAC
US_RR GCCTGTCTTCTCTACACCAAACACATCGTGCACAAGCTGCCCAACAATGTGATCTACAAC
****

AmFV_221 ATCGTACGTGAAGCCGTACATGCTGAGTCCATATTCTTTGGTCATGCACTCCGAAATGGA
US_RR ATCGTACGTGAAGCCGTACATGCTGAGTCCATCTTCTTTGGTCATGCACTCCGAAACGGA
********************************* ********************** ***

AmFV_221 CCGGTCGGTGAGCTCAGCCAACACAACATGATGCAGTATGTTCGCTTTTGCGCGGACCGT
US_RR CCGGTCGGCGAGCTCAGCCAACACAACATGATGCAGTATGTTCGCTTTTGCGCGGACCGT
******** ***

AmFV_221 TTGCTCACGGCCCTTGATGTGCCACCTCTGTACTCGGCCACCAATCCGTTCGATTTCATG
US_RR TTGCTCACGGCCCTTGATGTGCCACCTCTGTATTCGGCCACCAATCCGTTCGATTTCATG
******************************** ***************************

AmFV_221 CACATGATCTCGTTGGATGGGAGAACCAACTTCTTTGAACGGCGCGTCGGCGAGTACCGA
US_RR CACATGATCTCGTTGGATGGGAGAACCAACTTCTTTGAACGGCGCGTCGGCGAGTACCGA
****

AmFV_221 CAGAGTCGTGTTCTAACCGCAAACGAACGCGAGTACGACGAGTCGTTTGGTCTTGACTAC

```
US_RR           CAGAGTCGTGTTCTAACCGCAAACGAACGCGAGTACGACGAGTCGTTTGGTCTTGACTAC
                ************************************************************

AmFV_221        GATTTTTAG
US_RR           GATTTTTAG
                *********
```
