# Supplementary figures and images for: The Apis mellifera Filamentous Virus Genome
Source: Viruses. 2015 Jul 9;7(7):3798–815. doi: 10.3390/v7072798 (PMC4517127; doi:10.3390/v7072798)

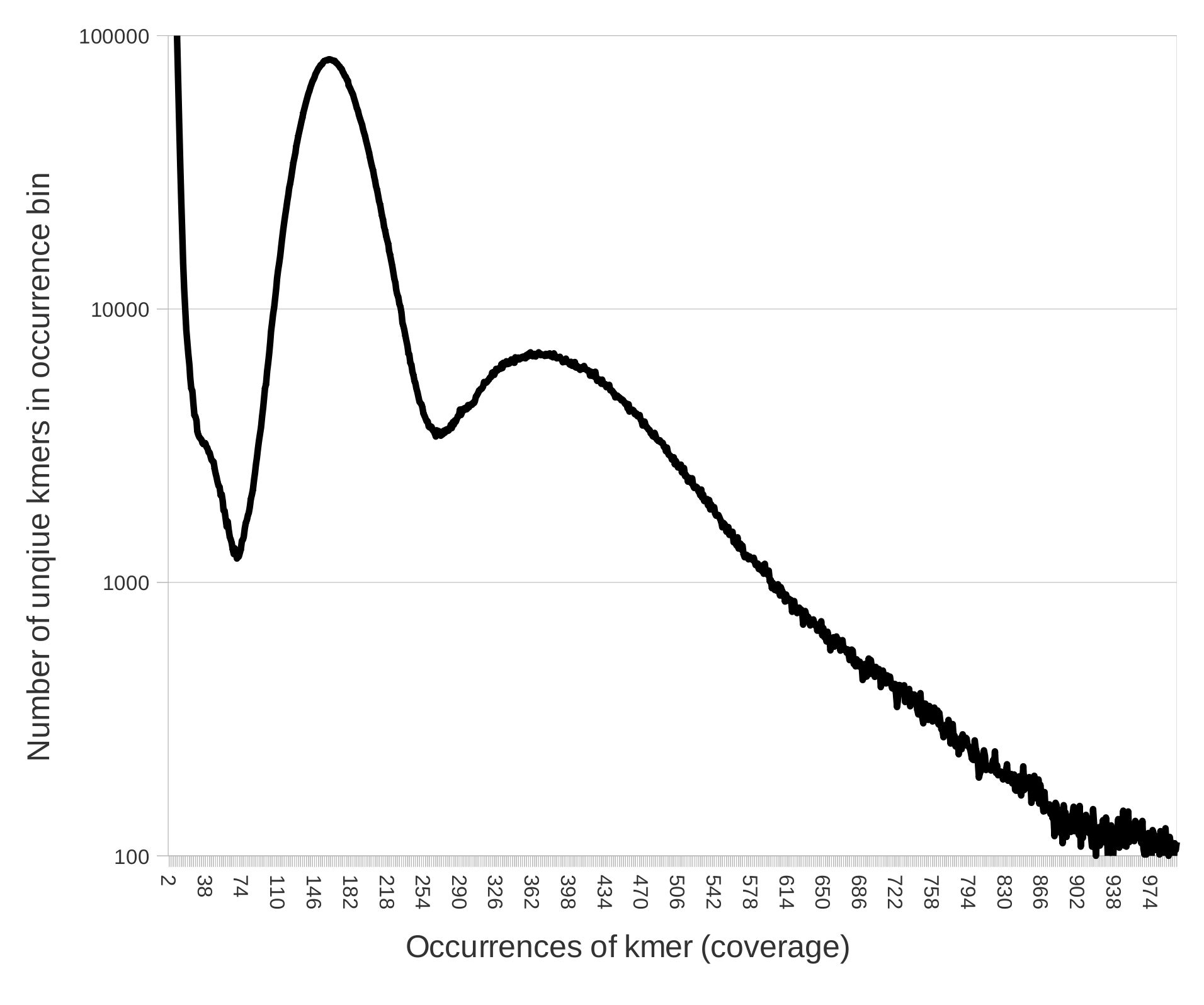

Supplement: Supplementary file 1 [file viruses-07-02798-s001.zip › Supp_File2a.jpg]
